# Non-linear association between weight-adjusted-waist index and obstructive sleep apnea: a cross-sectional study from the NHANES (2005–2008 to 2015–2020)

**DOI:** 10.3389/fpubh.2025.1546597

**Published:** 2025-03-25

**Authors:** Shu Miao, Qin Ben, Cai Song, Yashi Zhou, Bingjie Xie, Juxiang Peng, Jukun Song

**Affiliations:** ^1^School of Stomatology, Guizhou Medical University, Guiyang, China; ^2^Guiyang Hospital of Stomatology, Guiyang, China; ^3^School of Stomatology, Zunyi Medical University, Zunyi, China; ^4^Department of Oral and Maxillofacial Surgery, The Affiliated Stomatological Hospital of Guizhou Medical University, Guiyang, China

**Keywords:** weight-adjusted waist index, obstructive sleep apnea, obesity, NHANES, cross-sectional study

## Abstract

**Background:**

The principal objective of the present investigation is to undertake an in-depth exploration of the relationship that exists between the newly introduced weight-adjusted waist index (WWI), employed as a surrogate way for corpulence, and obstructive sleep apnea (OSA).

**Methods:**

Analysis using cross-sectional data from 11,545 NHANES participants across 2005–2008 and 2015–2020. Obesity via WWI (waist circumference over sqrt of body weight). OSA via 3 NHANES QnA items: monthly excessive sleepiness, weekly wheezing/snoring/breathing stoppage, weekly snoring. Relationships between WWI and OSA probed with weighted multivariate logistic regression and smoothed curve fitting. Also did subgroup, interaction tests and threshold effect analysis. Excluded those with incomplete WWI, OSA or hypertension data as they might have different health profiles. We excluded participants with incomplete data on WWI, OSA, or hypertation-related items, as those with missing data might have different health profiles.

**Results:**

The study, encompassing a cohort of 11,545 participants, revealed that 5,727 individuals were diagnosed with OSA. Upon conducting fully adjusted models, A positive relevance between WWI and OSA was established, with an odds ratio of 1.57 (95% *CI*: 1.44, 1.71), indicating a significant relationship. Notably, participants falling within the highest quartile of WWI exhibited a markedly heightened propensity for OSA, being 2.58 times more likely to suffer from it than those in the bottom quartile [*OR*: 2.58 (95% *CI*: 2.10, 3.17)]. Rigorous subgroup analyses and interaction tests further confirmed the robustness of this positive association across various subgroups, thereby affirming the consistency of the observed relationship. Additionally, a noteworthy non-linear association and saturation phenomenon were discerned between the WWI and OSA, demarcated by an inflection point at 11.70 cm/√kg.

**Conclusion:**

Our research has clearly shown a significant positive correlation, along with a saturation effect, between WWI and OSA in the American population. However, the cross-sectional design limits causal inference, and the exclusion of certain participants may affect the generalizability of the findings. Future longitudinal studies are needed to explore causality and address potential biases associated with participant exclusion, ultimately improving the broader applicability of the results.

## Introduction

The incidence of obstructive sleep apnea (OSA) is on the rise globally. Community studies from developed countries have shown that the prevalence of OSA in adult men can reach between 15 and 30%, while in adult women, it ranges from 5 to 15%. For example, a sleep cohort study in the US state of Wisconsin has shown a gradual increase in the prevalence of OSA over time. This trend indicates that the incidence of OSA is gradually increasing in different groups (1). OSA, a condition in which the upper respiratory tract becomes blocked during sleep, has become more common in recent years and has become an increasingly important public health issue. OSA can cause decreased oxygen saturation and disrupted sleep, leading to symptoms such as snoring, apnea, and excessive daytime sleepiness (2). The occurrence of OSA is closely related to the anatomy of the upper respiratory tract, involving the nasal cavity, nasopharynx, oropharynx, laryngeal pharynx, and larynx. Malformations or lesions in any of these structures can cause the airway to narrow or collapse, triggering apnea. Several studies have shown that anatomical abnormalities in the nasal cavity and oropharynx are linked positively to the severity of OSA (3, 4). In addition, obesity is a significant risk factor for the occurrence and development of OSA, which the academic community has widely confirmed (5). The occurrence of OSA is also closely related to neuromuscular factors, endocrine and metabolic disorders, and genetic factors (2, 6, 7).

OSA is not merely associated with a significant deterioration in patients' quality of life, but also exhibits a robust correlation with the incidence of various cardiovascular diseases, metabolic, as well as neurological conditions including coronary heart disease, hypertension, diabetes, and cerebrovascular incidents, which together constitute a significant threat to patient health (8, 9). Several studies have shown a correlation between the severity of OSA and the incidence of cancer, suggesting a potential causal relationship (10, 11). In addition, OSA is closely associated with the occurrence of psychiatric disorders, further expanding the scope of its clinical manifestations (12, 13). Obesity is acknowledged as a critical risk factor for OSA, and in particular, traditional measures of obesity, such as body mass index (BMI), are commonly utilized to predict an individual's susceptibility to OSA (14).

Although BMI is commonly used to assess obesity, it has inherent limitations, such as its inability to differentiate between adipose tissue and muscle mass and its failure to account for variations in body fat distribution (15). These limitations reduce BMI's accuracy in capturing the true nature of obesity, particularly in its relationship with conditions like OSA. In contrast, the Weight-Adjusted Waist Index (WWI) offers a more comprehensive and accurate assessment of obesity. WWI, derived from the quotient of waist circumference to the square root of body weight, better reflects central adiposity, which is a stronger predictor of metabolic and cardiovascular risks (16, 17). Additionally, WWI accounts for both abdominal fat and overall body weight, offering a more nuanced view of body fat distribution and obesity severity. This makes WWI a potentially more effective indicator for understanding the complex relationship between obesity and OSA, addressing the need for more precise measures in this field (18, 19). Despite this, research on the link between WWI and OSA is still scarce. To address this knowledge gap, this study conducted a cross-sectional analysis using well-designed National Health and Nutrition Examination Survey (NHANES) data from 2005–2008 to 2015–2020. NHANES is a large-scale, rigorously designed clinical registry with a wealth of follow-up data, offering a distinct advantage in examining the relationship between WWI and OSA. The primary aim of the study was to assess the association between WWI and OSA, suggesting that WWI could be an effective predictor of OSA. By leveraging NHANES resources, this investigation elucidates the nexus between WWI and OSA, offering potentially transformative implications for clinical practice.

## Methods

### Materials and methods data source

This cross-sectional study utilizes a publicly accessible dataset sourced from NHANES, available at https://wwwn.cdc.gov/nchs/nhanes/Default.aspx, thereby eliminating the necessity for additional approvals from an ethical review committee.

### Study population

**Figure 1:**
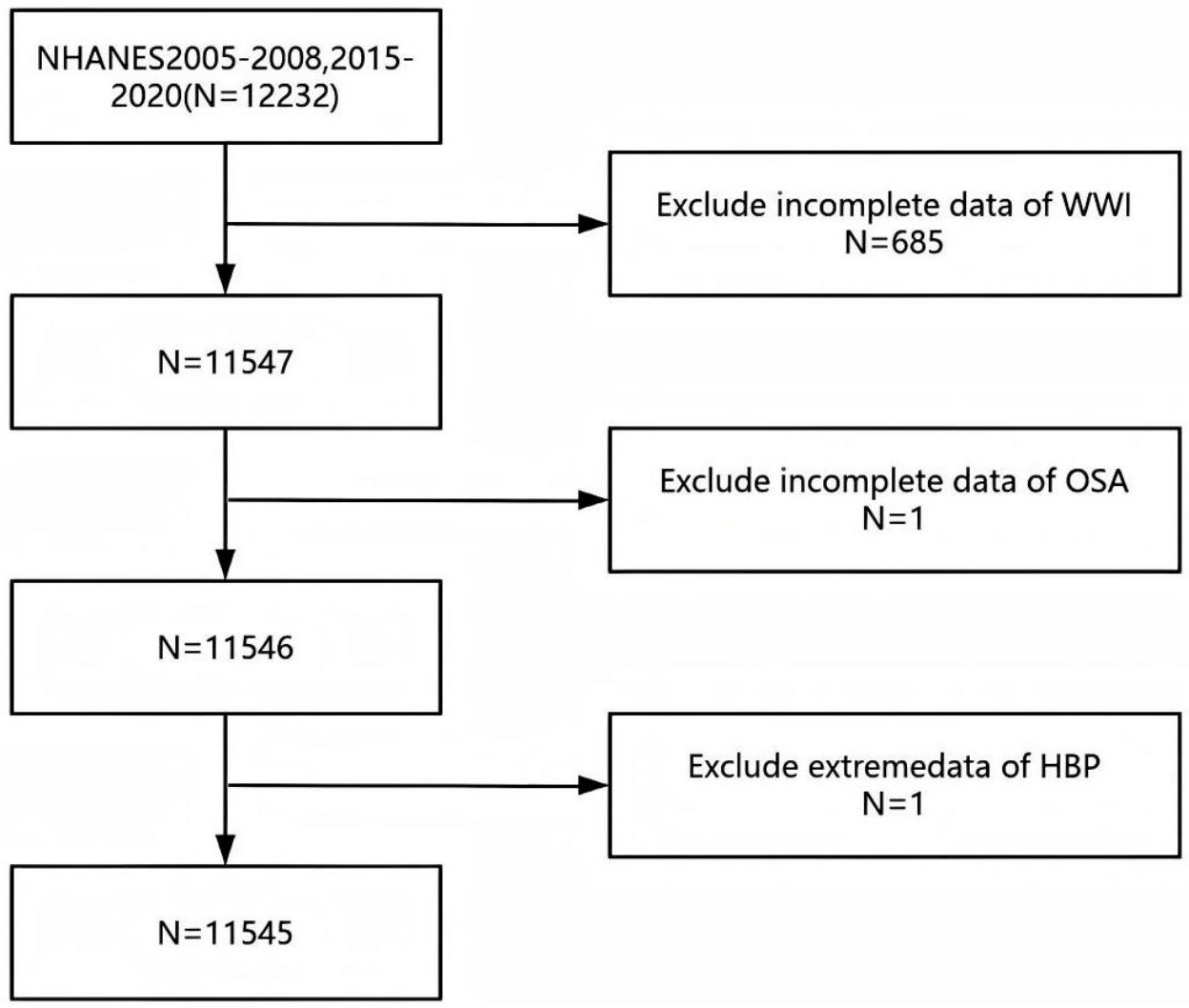
Flowchart of the study design and participants who were excluded from the study.

The dataset employed is derived from NHANES, a stratified, multistage probability sample survey conducted by the National Center for Health Statistics (NCHS) executes a pivotal survey, the National Health and Nutrition Examination Survey (NHANES), to assess the health and nutritional status across adult and pediatric demographics in the United States. NHANES, which boasts national representativeness, utilizes a meticulously detailed information collection methodology, as outlined on its website [http://www.cdc.gov/nchs/nhanes.htm; (20)]. Our research analyzed data from the NHANES survey cycles spanning 2005–2008, and 2015–2020, initially encompassing a cohort of 12,232 participants. However, after excluding individuals with incomplete data on WWI (*n* = 685) and those incomplete information on OSA and hypertension (*n* = 2), the final analysis sample comprised 11,545 participants (Figure 1). In our study, missing values for the covariates were categorized as a separate group. This was undertaken in order to reduce bias.

### Assessment of WWI

The WWI, an anthropometric measure was developed to assess body fat mass by normalizing waist circumference concerning weight and muscle mass to evaluate obesity (21). Certified health technicians, using the mobile examination center, gathered weight and body measurement data from participants. Participants were directed to don examination attire and stand unshod on a digital scale, arms positioned at their sides, with a forward gaze. This protocol adheres to the standardized methodologies for obtaining measurements (22). WC was determined using tape measures placed accurately at the junction of the mid-axillary line with a horizontal line that runs just superior to the lateral border of the right patella, following the standard protocol outlined in the guidelines (17). The calculation of WWI necessitated the implementation of the following formula: WC (cm) divided by weight (kg) squared root (23). Within our analytical framework, WWI was initially regarded as a continuous variable and subsequently stratified into quartiles to group participants for further exploration.

### Assessment of OSA

In accordance with earlier studies (24, 25), a diagnosis of OSA is conferred upon individuals who provide affirmative responses to at least one of the three diagnostic inquiries posed by NHANES, namely, ① the reporting of excessive daytime sleepiness occurring between 16 and 30 times per month, despite having achieved a minimum of 7 h of sleep per night during weekdays or weekday evenings ② experiencing wheezing, snoring, or cessation of breathing for three or more nights weekly; or ③ engaging in snoring for three or more nights weekly.

### Assessment of covariates

Drawing upon previous research findings, our study encompassed a wide spectrum of covariates that Certain demographic factors, encompassing age, gender, and ethnicity (categorized distinctly as Mexican American, Other Hispanic, Non-Hispanic White, Non-Hispanic Black, and Other Race), have the potential to be covariates that modulate the relationship between WWI and OSA; socioeconomic indicators including marital status, family income, and poverty-income ratio [PIR; (26)]. Educational attainment (27), lifestyle habits like alcohol consumption (28) and smoking status (29), sleep duration (30), medical conditions such as coronary heart disease [CHD, (28)], hypertension (31), diabetes (32), and Body Mass Index [BMI; (27)].

### Statistical analysis

Consistent with the methodological directives provided by the Centers for Disease Control and Prevention (CDC), a sophisticated multi-stage cluster sampling design was utilized in the statistical analysis, categorical variables were thoroughly assessed using *chi-square tests*, while continuous variables underwent meticulous examination through analysis of variance *(ANOVA)*. To ascertain the odds ratio (*OR*) with its corresponding 95% confidence interval (CI) that reflects the association between WWI and OSA, weighted multivariate Logit models—designated as Models I, II, and III—were implemented. Beginning with Model I, which was not controlled for any covariates, we then applied Model II, accounting for age, gender, and race. Finally, Model III was extended to include further controls for gender, age, race, education, marital status, PIR, smoking status, alcohol consumption, hypertension, diabetes, coronary heart disease, and sleep duration. To investigate potential disparities among different population segments, subgroup analyses and interaction assessments were performed. Additionally, the study explored the potential for a non-linear relation between OSA and the WWI index through the application of smoothed curve fitting techniques. The WWI was reclassified into quartile categories to facilitate a sensitivity analysis. The entire statistical analysis process was facilitated by the utilization of EmpowerStats 4.2 software (accessible at http://www.empowerstats.com) and Stata software (version 18), with statistical significance attributed to *P* < 0.05 in interpreting the results.

## Results

### Baseline traits of participants

The survey, encompassing a total of 11,545 participants, comprised a demographic breakdown of 49.00% men and 51.00% women, with ethnic distributions being 9.94% non-Hispanic white, 15.82% non-Hispanic black, 23.32% Mexican-American, 39.44% other Hispanic, and 11.49% belonging to other racial groups. The quartiles for WWI were demarcated as follows: Quartile 1, respectively, spanning values from 8.04 to 10.47; Quartile 2, encompassing values from 10.47 to 11.05; Quartile 3, ranging between 11.05 and 11.65; and Quartile 4, comprising values from 11.65 to 14.41. A substantial proportion of 49.61% of the participants were diagnosed with OSA overall, with a notable increase in OSA prevalence observed across higher WWI quartiles (Q1: 37.80%, Q2: 50.76%, Q3: 53.85%, Q4: 56.01%; *p* < 0.001). Individuals within the highest WWI quartile exhibited a profile characterized by older age, female gender, other Hispanic ethnicity, lower socioeconomic status, marital statuses of divorced, widowed, or separated, lower educational attainment, reduced alcohol consumption, former smoking status, higher body weight, elevated WC, increased BMI, and a greater propensity for diabetes, hypertension, and coronary heart disease relative to individuals in the lowest quartile (*p* < 0.001). Although sleep duration exhibited variability across different WWI zones (*p* < 0.001), these differences were relatively minor in magnitude (Table 1). The prevalence of OSA was 94.33% in the included group and 94.46% in the excluded group. There was no significant difference in OSA prevalence between the two groups (χ^2^ = 0.097, *P* = 0.755; Supplementary Table 1).

**Table 1:** Baseline participant characteristics.

		**Q1**	**Q2**	**Q3**	**Q4**		
**WWI**	**Overall**	**8.04–10.47**	**10.47–11.05**	**11.05–11.65**	**11.65–14.41**	***P*** **-value**	***P*** **-value** ^*^
**WC (cm**)	99.39 ± 16.62	84.59 ± 10.66	96.32± 11.84	103.44 ± 13.05	113.21 ± 15.65	<0.001	<0.001
**Weight (kg)**	81.86± 21.52	73.39 ± 16.97	81.18± 19.80	84.48 ± 21.35	88.38± 24.38	<0.001	<0.001
**Race (%)**						<0.001	-
Non-Hispanic Black	1,826 (15.82%)	288 (9.98%)		545 (18.88%)	530 (18.36%)		
Non-Hispanic White	1,148 (9.94%)	194 (6.72%)	296 (10.26%)	308 (10.67%)	350 (12.12%)		
Other Hispanic	4,553 (39.44%)	1,070 (37.08%)	1,134 (39.29%)	1,112 (38.53%)	1,237 (42.85%)		
Mexican American	2,692 (23.32%)	929 (32.19%)	628 (21.76%)	615 (21.31%)	520 (18.01%)		
Other races	1,326 (11.49%)	405 (14.03%)	365 (12.65%)	306 (10.60%)	250 (8.66%)		
**Sex (%)**						<0.001	-
Male	5,657 (49.00%)	1,708 (59.18%)	1,593 (55.20%)	1,374 (47.61%)	982 (34.02%)		
Female	5,888 (51.00%)	1,178 (40.82%)	1,293 (44.80%)	1,512 (52.39%)	1,905 (65.99%)		
**AGE (%)**						<0.001	-
<44	4,737 (41.03%)	2,022 (70.06%)	1,268 (43.94%)	870 (30.15%)	577 (19.99%)		
[44, 60]	2,949 (25.54%)	584 (20.24%)	903 (31.29%)	823 (28.52%)	639 (22.13%)		
≥60	3,859 (33.43%)	280 (9.70%)	715 (24.78%)	1,193 (41.34%)	1,671 (57.88%)		
**Education level (%)**						<0.001	-
<High school	2,707 (23.45%)	424 (14.69%)	581 (20.13%)	769 (26.65%)	933 (32.32%)		
High school	2,682 (23.23%)	592 (20.51%)	658 (22.80%)	728 (25.23%)	704 (24.39%)		
>High school	5,849 (50.66%)	1,662 (57.59%)	1,597 (55.34%)	1,359 (47.09%)	1,231 (42.64%)		
**Marital status (%)**						<0.001	-
Married/living with Partner	6,799 (58.89%)	1,496 (51.84%)	1,836 (63.62%)	1,856 (64.31%)	1,611 (55.80%)		
Widowed/divorced/separated	2,462 (21.33%)	350 (12.13%)	534 (18.50%)	647 (22.42%)	931 (32.25%)		
Never married	1,978 (17.13%)	831 (28.79%)	467 (16.18%)	353 (12.23%)	327 (11.33%)		
**PIR (%)**						<0.001	-
<1	2,079 (18.01%)	463 (16.04%)	463 (16.04%)	506 (17.53%)	647 (22.41%)		
[1, 3]	4,463 (38.66%)	992 (34.37%)	1,075 (37.25%)	1,170 (40.54%)	1,226 (42.47%)		
≥3	3,853 (33.37%)	1,159 (40.16%)	1,084 (37.56%)	921 (31.91%)	689 (23.87%)		
**BMI (%)**						<0.001	-
≤25	3,390 (29.40%)	1,711 (59.29%)	845 (29.33%)	541 (18.77%)	293 (10.17%)		
[25, 30]	3,783 (32.80%)	820 (28.41%)	1,121 (38.91%)	1,045 (36.25%)	797 (27.65%)		
>30	4,359 (37.80%)	355 (12.30%)	915 (31.76%)	1,297 (44.99%)	1,792 (62.18%)		
**Alcohol consumption (%)**						<0.001	-
Never	2,132 (18.47%)	292 (10.12%)	452 (15.66%)	615 (21.31%)	773 (26.78%)		
Moderate	6,223 (53.90%)	1,820 (63.06%)	1,681 (58.25%)	1,462 (50.66%)	1,260 (43.64%)		
Heavy	1,096 (9.49%)	313 (10.85%)	310 (10.74%)	294 (10.19%)	179 (6.20%)		
**Smoking (%)**						<0.001	-
Never	6,402 (55.45%)	1,733 (60.05%)	1,603 (55.54%)	1,500 (51.98%)	1,566 (54.24%)		
Former	2,784 (24.11%)	456 (15.80%)	670 (23.22%)	830 (28.76%)	828 (28.68%)		
Now	2,346 (20.32%)	694 (24.05%)	609 (21.10%)	555 (19.23%)	488 (16.90%)		
**HBP (%)**						<0.001	-
No	6,757 (58.53%)	2,325 (80.56%)	1,812 (62.79%)	1,521 (52.70%)	1,099 (38.07%)		
Yes	4,788 (41.47%)	561 (19.44%)	1,074 (37.21%)	1,365 (47.30%)	1,788 (61.93%)		
**Diabetes (%)**						<0.001	-
No	9,416 (81.56%)	2,763 (95.74%)	2,528 (87.60%)	2,260 (78.31%)	1,865 (64.60%)		
Yes	2,129 (18.44%)	123 (4.26%)	358 (12.41%)	626 (21.69%)	1,022 (35.40%)		
**CHD (%)**						<0.001	-
No	10,732 (92.96%)	2,654 (91.96%)	2,750 (95.29%)	2,710 (93.90%)	2,618 (90.68%)		
Yes	465 (4.03%)	22 (0.76%)	84 (2.91%)	130 (4.51%)	229 (7.93%)		
**Sleep duration (%)**						<0.001	-
<7	3,509 (30.39%)	902 (31.25%)	915 (31.71%)	862 (29.87%)	830 (28.75%)		
[7, 9]	6,048 (52.39%)	1,535 (53.19%)	1,542 (53.43%)	1,522 (52.74%)	1,449 (50.19%)		
≥9	1,940 (16.80%)	443 (15.35%)	419 (14.52%)	487 (16.88%)	591 (20.47%)		
**OSA (%)**						<0.001	-
No	5,818 (50.39%)	1,795 (62.20%)	1,421 (49.24%)	1,332 (46.15%)	1,270 (43.99%)		
Yes	5,727 (49.61%)	1,091 (37.80%)	1,465 (50.76%)	1554 (53.85%)	1,617 (56.01%)		

Values are expressed as *N* (%) for categorical variables. Continuous variable expressed as mean ± standard deviation; *P*-value was calculated using the weighted chi-squared test for categorical variables; In the case of continuous variables, the Kruskal Wallis rank sum test is used, and in the case of counting variables with a theoretical number <10, the Fisher exact probability test is used; Q, quartile; In sensitivity analysis, WWI was converted from a continuous variable to a categorical variable (quartiles); Numbers that do not add up to 100% are attributable to missing data. Bold font includes incorporated variables.

* Fisher exact probability test.

### The correlation between WWI and OSA

The relationship between WWI and OSA is depicted in Table 2, indicating a positive correlation between increased WWI exposure and the likelihood of OSA. Across Models 1, 2, and 3, a consistent positive correlation between OSA and WWI was observed. Notably, after comprehensive adjustments in Model 3, individuals with an elevated WWI showed an increased risk of OSA by a factor of 1.57, the analysis revealed a statistically significant correlation, with an odds ratio (*OR*) of 1.57 and a 95% confidence interval (*CI*) extending from 1.44 to 1.71 (Model 3: *OR* = 1.57, 95% *CI*: 1.44 ~ 1.71). When WWI was categorized into quartiles, the statistical significance of the correlation persisted. Notably, participants in the highest WWI quartile exhibited a substantial 2.58-fold increased risk of OSA relative to individuals in the lowest quartile, yielding an *OR* of 1.59 with a 95% *CI* extending from 1.36 to 1.85, and a p-value for the trend that was <0.0001, indicating a robust and statistically significant association (*OR* = 1.59, 95 % *CI*: 1.36 to 1.85; *P* for trend < 0.0001).

**Table 2:** The association between WWI and OSA.

**OR (95%CI)** ***P*****-value**			
	**Model 1**	**Model 2**	**Model 3**
WWI Continuous	1.45 (1.37, 1.55) <0.0001	1.65 (1.52, 1.78) <0.0001	1.57 (1.44, 1.71) <0.0001
**WWI quartile**			
Q1	Ref.	Ref.	Ref.
Q2	1.73 (1.50, 1.99) <0.0001	1.734 (1.49, 2.01) <0.0001	1.59 (1.36, 1.85) <0.0001
Q3	2.11 (1.79, 2.49) <0.0001	2.31 (1.96, 2.73) <0.0001	2.09 (1.76, 2.47) <0.0001
Q4	2.26 (1.96, 2.61) <0.0001	2.89 (2.40, 3.48) <0.0001	2.58 (2.10, 3.17) <0.0001
*P* for trend	<0.0001	<0.0001	<0.0001

Model 1 Adjust for: none; Model 2 Adjust for: age; sex; race; Model 3 Adjust for: sex; age; race; education level; marital status; ratio of family income to poverty; alcohol consumption; smoking; hypertension; diabetes; coronary heart disease; sleep duration.

### Analysis of curve fitting and threshold effects

**Figure 2:**
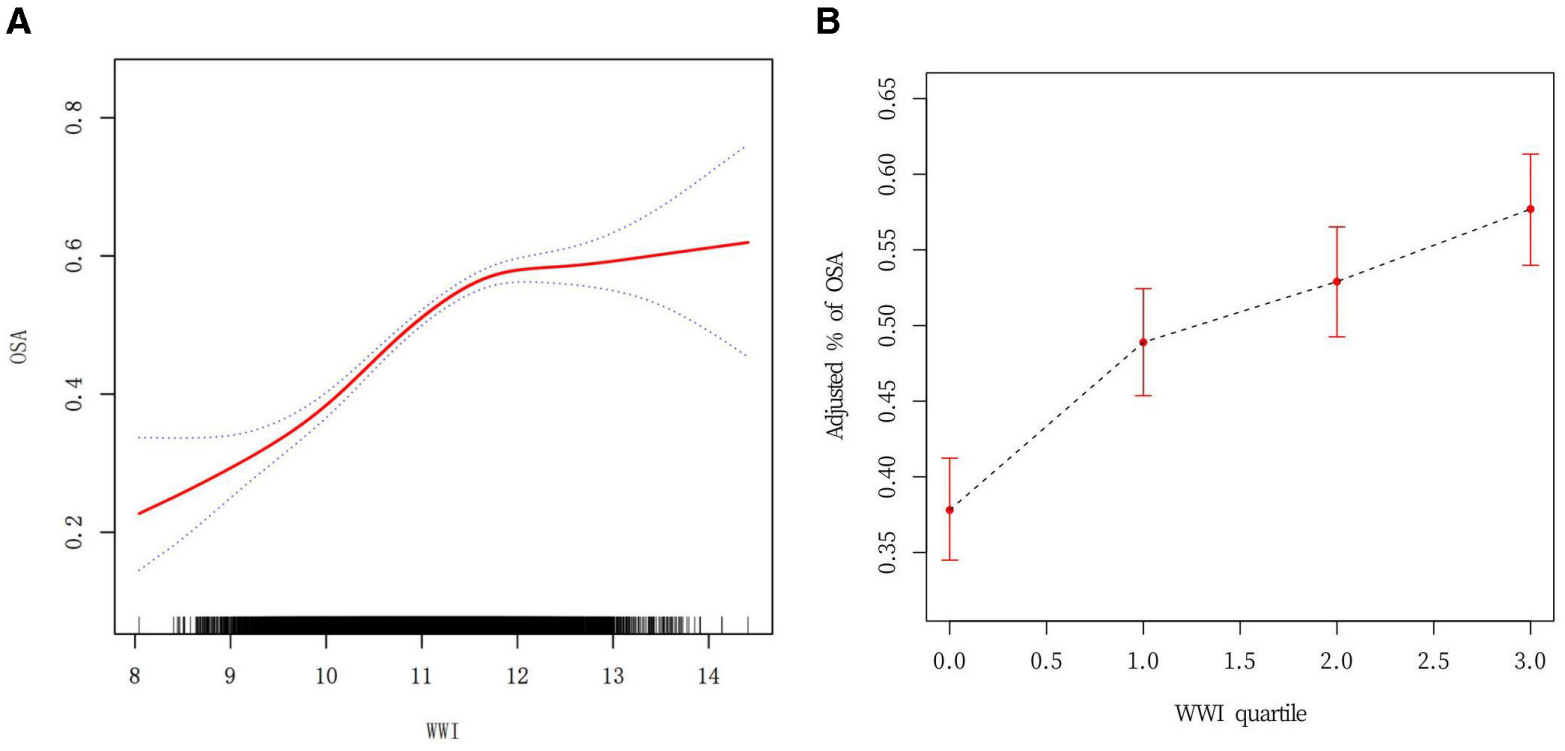
**(A)** Curve fitting for WWI (Continuous) vs. OSA. Smoothed curve fitting of WWI vs. OSA. Detection of non-linear relationship between WWI and OSA by logistic regression modeling. The red solid line represents the smoothed curve fit between the variables. The blue dashed line represents the 95% CI of the fitted results. **(B)** Curve fitting for WWI (quartile) vs. OSA.

The analysis revealed a non-linear association between WWI and the incidence of OSA (Figure 2A). Sensitivity analyses were conducted by transforming WWI into categorical variables, and the results showed a positive correlation as the adjusted OSA percentage gradually increased with the increase in WWI (Figure 2B). Upon closer examination of the threshold effect, a key inflection point was identified at a WWI value of 11.70 (Table 3). When WWI was below this threshold, the likelihood of developing OSA increased by 60% for each unit increment (*OR* = 1.60; 95% *CI*: 1.49–1.72; *p* < 0.0001). In contrast, for WWI values above the threshold, no statistically significant relationship was discovered (*OR* = 0.97; 95% *CI*: 0.84–1.13; *p* = 0.69). The threshold's statistical significance was substantiated by the likelihood ratio test, yielding a *p*-value significantly below the 0.001 threshold.

**Table 3:** The threshold effect analysis of the WWI on OSA risk.

**Outcome**	**OSA**
**Model I**	
A linear effect.	1.42(1.35, 1.50) <0.0001
**Model II**	
WWI turning point (K)	11.70
<K, effect 1	1.60 (1.49, 1.72) <0.0001
> K, effect 2	0.97 (0.84, 1.13) 0.69
The effect difference between 2 and 1	0.61 (0.50, 0.73) <0.0001
The predicted value at the turning point of the equation.	0.37 (0.31, 0.44)
Log-likelihood ratio	<0.001

Adjust for: sex; age; race; education level; marital status; ratio of family income to poverty; alcohol consumption; smoking; hypertension; diabetes; coronary heart disease; sleep duration.

### Subgroup analysis

**Figure 3:**
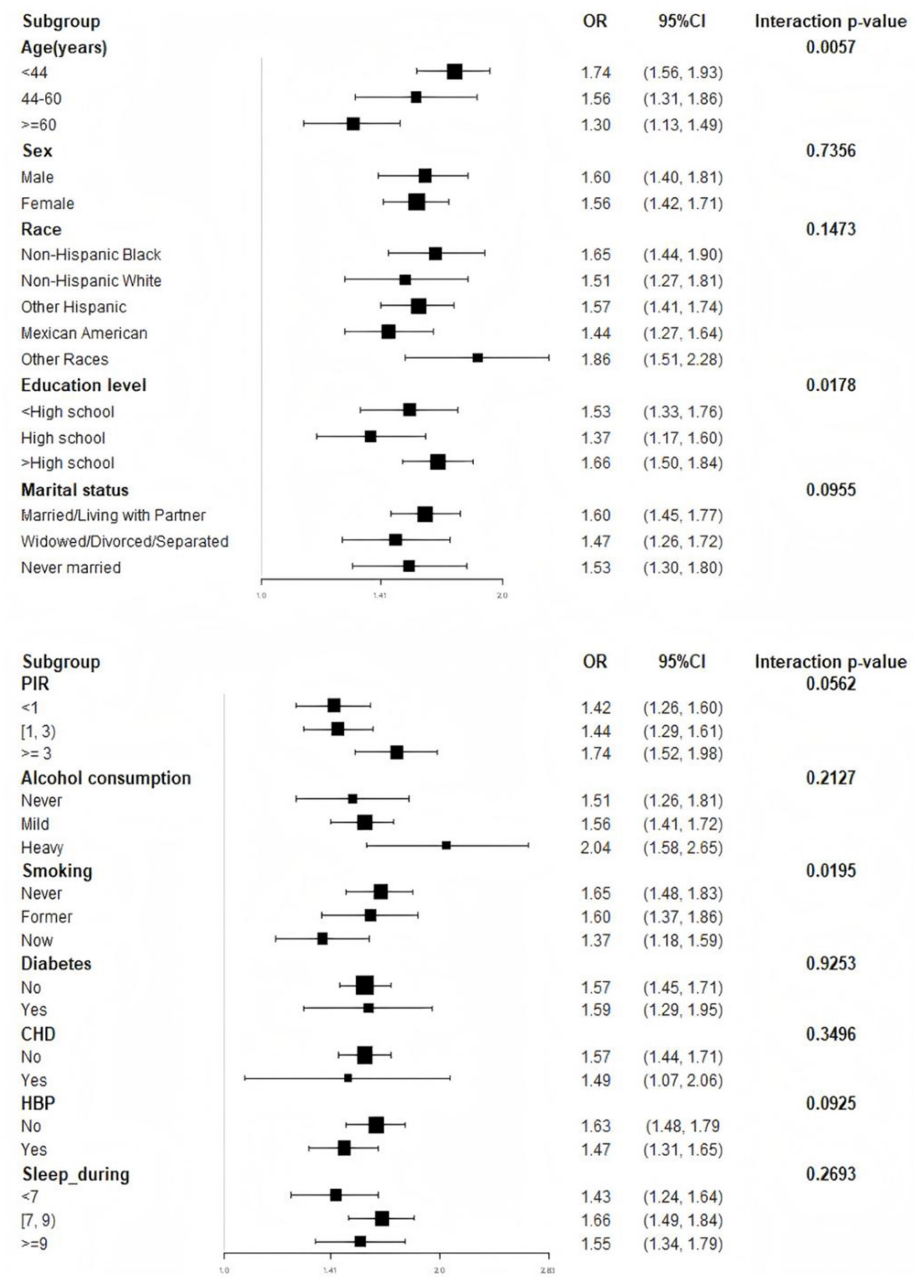
Subgroup analysis and interaction for the association between WWI and OSA.

A comprehensive subgroup analysis was conducted to evaluate the strength and consistency of the association between WWI and OSA, with stratification by all covariates. An interaction test was performed for age, gender, race, education level, marital status, PIR, alcohol consumption, smoking, hypertension, diabetes, CHD, and sleep duration. The results revealed statistically significant interactions for age (*p* = 0.0057), education level (*p* = 0.0178), and smoking (*p* = 0.0195), suggesting that these factors may modify the relationship between WWI and OSA. Notably, variability in the relationship by race was observed, with non-Hispanic Blacks (OR = 1.65, 95% CI: 1.44, 1.90) and individuals from other racial groups (OR = 1.86, 95% CI: 1.51, 2.28) showing higher odds ratios compared to non-Hispanic Whites (OR = 1.51, 95% CI: 1.27, 1.81) and Mexican Americans (OR = 1.44, 95% CI: 1.27, 1.64). However, no significant interactions were found for other covariates, including sex (*p* = 0.7356), marital status (*p* = 0.0955), PIR (*p* = 0.0562), alcohol consumption (*p* = 0.2127), CHD (*p* = 0.3496), hypertension (*p* = 0.0925), diabetes (*p* = 0.9253), or sleep duration (*p* = 0.2693), indicating that the relationship between WWI and OSA was not strongly dependent on these factors (Figure 3). Furthermore, we developed curve-fitting analyses for the three covariates that exhibited significant interactions (Supplementary Figure 1).

## Discussion

This study was a cross-sectional study involving 11,545 non-hospitalized residents of the United States to investigate the relationship between WWI and OSA. The findings revealed a notable correlation where an escalation in WWI was considerably linked to a higher prevalence of OSA, and the association was statistically significant. This consistency was maintained regardless of the WWI being categorized into quartiles (Q1–Q4). Additional subgroup analyses and assessments of interactions confirmed that the significant positive association persisted across the entire spectrum of subgroups, including factors such as sex, age, race, education, PIR, marital status, smoking behavior, alcohol intake, hypertension, diabetes, coronary heart disease, and sleep duration. It is important to note that the interaction p-values for age groups, education levels, and smoking behaviors were found to be statistically significant *(p* ≤ 0.05). This indicates that the associations between WWI and OSA may vary across these subgroups, suggesting potential modifiers of the WWI-OSA relationship. Notably, the study also found a non-linear relationship in which the association with OSA risk decreased when WWI exceeded 11.70. This critical threshold may provide a new perspective for future risk assessment and intervention strategies. The results of this study suggest that WWI can be used as a predictor of OSA incidence and suggest that obesity management as measured by WWI may help predict OSA risk. The biological mechanisms behind this may include changes in fat distribution, development of metabolic syndrome, and increased insulin resistance (33, 34). These findings suggest that the threshold effect of WWI may be associated with changes in insulin sensitivity in adipose tissue, which may further influence the relationship with OSA. However, the practical implications of the WWI threshold value (11.70 cm/√kg) remain to be fully explored. Future studies are needed to examine its potential role in clinical decision-making, including its utility in risk stratification, early detection, and the development of targeted disease management strategies. In the context of data analysis, the inclusion of missing values for covariates as a distinct group is primarily motivated by the desire to preserve the integrity of the data and to mitigate potential bias. Missing values for categorical variables possess the potential to convey informative content in their own right; the direct replacement or removal of these missing values may result in the introduction of bias or the masking of underlying patterns (35). Moreover, the segregation of missing values facilitates the avoidance of distributional imbalances that may be introduced by the application of the filling method, thereby providing a more robust foundation for subsequent analyses. This approach has been widely adopted in analogous studies that have dealt with missing values for categorical variables. For instance, some studies have recommended treating missing values as a distinct category in order to more accurately reflect the actual structure of the data (36).

Growing research supports the notion that obesity plays a substantial role as a risk factor in the development of OSA (8, 14). For example, Leeba Rezaie et al. analyzed data from 251 participants and concluded that a higher BMI could help non-specialist healthcare providers identify individuals at increased risk for OSA (37). Similarly, a prospective study by Gruber et al., involving 38 patients with OSA and 41 controls, found that higher BMI and waist circumference increased the likelihood of developing OSA (38). In addition, in a cross-sectional study of 4,588 participants aged 20 years and older in the United States, Xingru Meng et al. found that the risk of OSA in the higher BMI quartile group was 3.42 times higher than that in the lower quartile group (39). This study compared OSA prevalence and demographic traits between included and excluded participants. No significant difference in OSA prevalence was found, indicating that inclusion criteria did not bias OSA assessment. However, differences in demographics such as sex, age, education, marital status, income, alcohol use, hypertension, diabetes, and heart disease were noted, which may limit the findings' generalizability. Future research should aim to further explore these differences and their implications for the prevention and management of OSA.

WWI, introduced as an innovative obesity metric, integrates the advantages of WC and diminishes the correlation with BMI, allowing for a more accurate description of centripetal obesity independent of weight. In addition, WWI provides an assessment of fat and muscle mass, surpassing the constraints of conventional BMI criteria (40, 41). It is worth noting that, compared with other anthropometric indicators such as waist-height ratio, BMI, and waist circumference, WWI is more significantly correlated with sarcopenic obesity in older adults (42). Thus, as a more comprehensive and accurate obesity assessment tool, WWI is expected to provide greater insight into the relationship between obesity and OSA (41).

Recent studies further consolidate the advantage of WWI as a predictor of disease compared to BMI and WC (22, 43). Evidence from WWI suggests that WWI offers a more precise and targeted evaluation of central adiposity compared to other fat distribution metrics like the waist-to-hip ratio (WHR), and its utility is consistent across various ethnic groups and populations. WWI has shown excellent stability and reliability, especially in cross-ethnic or multi-center studies (44). Therefore, WWI is recognized for its simple calculation, cost-effectiveness, and excellent predictive power in disease risk assessment, and it possesses superior predictive accuracy for disease risk, warranting the attention of healthcare providers. Multiple mechanisms have been put forth to account for the observed positive association between WWI and OSA, including changes in upper airway structure and function, enhanced airway collapse, reduced resting lung volume, and disruption of the interaction between respiratory drive and load compensation (45). Specifically, obesity, particularly central obesity, increases susceptibility to sleep apnea by increasing the mechanical load on the upper respiratory tract and reducing compensatory neuromuscular responses (46, 47). These findings further reinforce the role of WWI as a critical indicator for understanding and predicting OSA risk and highlight its broader impact on disease prediction.

The practical importance of this research is underscored by the identification of WWI as a potential predictive biomarker for assessing the risk of OSA in adult individuals. Specifically, WWI was superior to conventional fat parameters in predicting OSA risk, providing a more robust risk assessment and enabling clinicians to incorporate WWI measurements into routine assessments to identify individuals at risk for OSA more precisely. In addition, recognizing WWI's role as a prognostic biomarker for OSA may enable medical professionals to develop tailored interventions that not only address obesity more effectively but also mitigate the risk of OSA.

WWI's predictive power is enhanced by its ability to more accurately capture centripetal obesity, independent of overall body weight, offering a significant advantage over traditional measures like BMI and waist-to-hip ratio (WHR). Unlike BMI, which fails to differentiate between adipose tissue and muscle mass and does not account for body fat distribution, WWI provides a more nuanced assessment of obesity-related risks. Additionally, WWI's ability to reflect abdominal fat distribution—an important factor for metabolic and cardiovascular health—makes it particularly valuable in identifying individuals at higher risk for conditions like OSA. Its utility across various ethnic groups and populations further emphasizes its potential as a reliable tool for public health assessments and disease risk prediction, addressing the limitations of BMI and WHR in diverse cohorts (48).

### Study strengths and limitations

This study explored the association between WWI and OSA through a cross-sectional examination, using a representative sample size to offer a new perspective on this relationship. While we applied multiple adjustment models and conducted subgroup analyses to enhance our understanding of population-specific differences, it is important to note that the cross-sectional design limits our ability to infer causality. Additionally, the reliance on self-reported data and the exclusion of certain covariates may introduce bias or affect the generalizability of the findings. Despite these limitations, our in-depth subgroup analyses revealed varying patterns of association, highlighting the complexity of the relationship between WWI and OSA across different cohorts. These findings provide valuable insights that could inform targeted interventions and public health policies, though further research is needed to confirm these results and address the limitations identified.

Although this study provides valuable insights, several limitations should be considered. First, the cross-sectional design of the study precludes the establishment of causal relationships, limiting the ability to infer directionality between WWI and OSA. Second, the data were sourced from the NHANES database, which, while widely used and validated in previous studies, may still be subject to selection bias and measurement inaccuracies (49, 50). The reliance on self-reported OSA diagnosis, in particular, introduces the possibility of recall bias, as participants may not accurately report their symptoms or may underreport or overreport them. While questionnaire-based assessments are common in large-scale surveys, they lack the precision of objective diagnostic methods like polysomnography. Future studies should aim to validate self-reported OSA assessments against objective measures to enhance the reliability of findings. Moreover, generalisability of results may be limited by different ethnic and socio-economic groups. Additionally, while we adjusted for several confounders, other potential confounders, such as physical activity and dietary habits, were not included and may have influenced the results. Physical activity and dietary habits are well-established factors affecting both obesity and sleep quality, and their exclusion may have resulted in an incomplete analysis. Lastly, participant exclusions may have introduced bias, particularly among subgroups with high WWI or severe OSA, which could affect the generalizability of the findings. Future studies would benefit from employing prospective designs and optimizing data collection to address these limitations and improve the accuracy and generalizability of the results.

## Conclusion

Our findings suggest a positive correlation between WWI and OSA, with a notable trend observed among U.S. adults. While these results are promising, it is important to recognize that this study is exploratory in nature, and the conclusions should be interpreted with caution due to the limitations inherent in the cross-sectional design, reliance on self-reported data, and potential biases from missing covariates. Although WWI appears to be a valid anthropometric indicator for predicting OSA, further research, including longitudinal studies, is essential to establish causality and explore its clinical applications in preventing and treating OSA. This study lays the groundwork for future investigations into WWI's role in OSA, and we hope it will encourage further research in this area.

## Data Availability

Publicly available datasets were analyzed in this study. This data can be found at: https://www.cdc.gov/nchs/nhanes/index.htm.

## References

[1] Young T Palta M Dempsey J Skatrud J Weber S Badr S. The occurrence of sleep-disordered breathing among middle-aged adults. N Engl J Med. (1993) 328:1230–5. 10.1056/NEJM199304293281704 8464434

[2] Jordan AS McSharry DG Malhotra A. Adult obstructive sleep apnoea. Lancet. (2014) 383:736–47. 10.1016/S0140-6736(13)60734-5 23910433 PMC3909558

[3] Young T Peppard PE Gottlieb DJ. Epidemiology of obstructive sleep apnea: a population health perspective. Am J Respir Crit Care Med. (2002) 165:1217–39. 10.1164/rccm.2109080 11991871

[4] Li KK Riley RW Powell NB Guilleminault C. Maxillomandibular advancement for persistent obstructive sleep apnea after phase I surgery in patients without maxillomandibular deficiency. Laryngoscope. (2000) 110:1684–8. 10.1097/00005537-200010000-00021 11037825

[5] Peppard PE Young T Palta M Dempsey J Skatrud J. Longitudinal study of moderate weight change and sleep-disordered breathing. JAMA. (2000) 284:3015–21. 10.1001/jama.284.23.3015 11122588

[6] Kimoff RJ Sforza E Champagne V Ofiara L Gendron D. Upper airway sensation in snoring and obstructive sleep apnea. Am J Respir Crit Care Med. (2001) 164:250–5. 10.1164/ajrccm.164.2.2010012 11463596

[7] Palmer LJ Buxbaum SG Larkin EK Patel SR Elston RC Tishler PV. Whole genome scan for obstructive sleep apnea and obesity in African-American families. Am J Respir Crit Care Med. (2004) 169:1314–21. 10.1164/rccm.200304-493OC 15070816

[8] Drager LF Togeiro SM Polotsky VY Lorenzi-Filho G. Obstructive sleep apnea: a cardiometabolic risk in obesity and the metabolic syndrome. J Am Coll Cardiol. (2013) 62:569–76. 10.1016/j.jacc.2013.05.045 23770180 PMC4461232

[9] Meszaros M Bikov A. Obstructive sleep apnoea and lipid metabolism: the summary of evidence and future perspectives in the pathophysiology of OSA-associated dyslipidaemia. Biomedicines. (2022) 10:2754. 10.3390/biomedicines10112754 36359273 PMC9687681

[10] Martínez-García M Campos-Rodriguez F Barbé F. Cancer and OSA: current evidence from human studies. Chest. (2016) 150:451–63. 10.1016/j.chest.2016.04.029 27164292

[11] Marriott RJ Singh B McArdle N Darcey E King S Bond-Smith D. Does OSA increase risk for cancer? Chest. (2023) 164:1042–56. 10.1016/j.chest.2023.04.043 37150506

[12] Gupta MA Simpson FC. Obstructive sleep apnea and psychiatric disorders: a systematic review. J Clin Sleep Med. (2015) 11:165–75. 10.5664/jcsm.4466 25406268 PMC4298774

[13] Liu H Wang X Feng H Zhou S Pan J Ouyang C. Obstructive sleep apnea and mental disorders: a bidirectional mendelian randomization study. BMC Psychiatry. (2024) 24:304. 10.1186/s12888-024-05754-8 38654235 PMC11040841

[14] Lyons MM Bhatt NY Pack AI Magalang UJ. Global burden of sleep-disordered breathing and its implications. Respirology. (2020) 25:690–702. 10.1111/resp.13838 32436658

[15] Donini LM Pinto A Giusti AM Lenzi A Poggiogalle E. Obesity or BMI paradox? Beneath the tip of the iceberg. Front Nutr. (2020) 7:53. 10.3389/fnut.2020.00053 32457915 PMC7221058

[16] Cao S Hu X Shao Y Wang Y Tang Y Ren S. Relationship between weight-adjusted-waist index and erectile dysfunction in the United State: results from NHANES 2001–2004. Front Endocrinol. (2023) 14:1128076. 10.3389/fendo.2023.1128076 37181040 PMC10167952

[17] Tao J Zhang Y Tan C Tan W. Associations between weight-adjusted waist index and fractures: a population-based study. J Orthop Surg Res. (2023) 18:290. 10.1186/s13018-023-03776-8 37038167 PMC10088134

[18] Qin Z Du D Li Y Chang K Yang Q Zhang Z. The association between weight-adjusted-waist index and abdominal aortic calcification in adults aged ≥ 40 years: results from NHANES 2013–2014. Sci Rep. (2022) 12:20354. 10.1038/s41598-022-24756-8 36437292 PMC9701694

[19] de Koning L Merchant AT Pogue J Anand SS. Waist circumference and waist-to-hip ratio as predictors of cardiovascular events: meta-regression analysis of prospective studies. Eur Heart J. (2007) 28:850–6. 10.1093/eurheartj/ehm026 17403720

[20] You Y Chen Y Yin J Zhang Z Zhang K Zhou J. Relationship between leisure-time physical activity and depressive symptoms under different levels of dietary inflammatory index. Front Nutr. (2022) 9:983511. 10.3389/fnut.2022.983511 36159493 PMC9490084

[21] Kim KJ Son S Kim KJ Kim SG Kim NH. Weight-adjusted waist as an integrated index for fat, muscle and bone health in adults. J Cachexia Sarcopenia Muscle. (2023) 14:2196–203. 10.1002/jcsm.13302 37550773 PMC10570086

[22] Xie F Xiao Y Li X Wu Y. Association between the weight-adjusted-waist index and abdominal aortic calcification in United States adults: results from the national health and nutrition examination survey 2013–2014. Front Cardiovasc Med. (2022) 9:948194. 10.3389/fcvm.2022.948194 36186965 PMC9515490

[23] Li M Yu X Zhang W Yin J Zhang L Luo G. The association between weight-adjusted-waist index and depression: results from NHANES 2005–2018. J Affect Disord. (2024) 347:299–305. 10.1016/j.jad.2023.11.073 38000467

[24] Scinicariello F Buser MC Feroe AG Attanasio R. Antimony and sleep-related disorders: NHANES 2005–2008. Environ Res. (2017) 156:247–52. 10.1016/j.envres.2017.03.036 28363141 PMC5685481

[25] Gu X Tang D Xuan Y Shen Y Lu LQ. Association between obstructive sleep apnea symptoms and gout in US population, a cross-sectional study. Sci Rep. (2023) 13:10192. 10.1038/s41598-023-36755-4 37353548 PMC10290056

[26] Cai S Li S Zhou Y Song J Peng J. The association between sedentary behavior and obstructive sleep apnea: a cross-sectional study from the NHANES (2007–2008 to 2015–2020). BMC Oral Health. (2024) 24:224. 10.1186/s12903-024-03960-0 38347492 PMC10863124

[27] Pan X Zhang X Wu X Zhao Y Li Y Chen Z. Association between non-high-density lipoprotein cholesterol to high-density lipoprotein cholesterol ratio and obstructive sleep apnea: a cross-sectional study from NHANES. Lipids Health Dis. (2024) 23:209. 10.1186/s12944-024-02195-w 38965618 PMC11223298

[28] Li E Ai F Liang C Chen Q Zhao Y Xu K. Latent profile analysis of depression in US adults with obstructive sleep apnea hypopnea syndrome. Front Psychiatry. (2024) 15:1398669. 10.3389/fpsyt.2024.1398669 38736623 PMC11082792

[29] Jiang S Wang Y Wang Z Zhang L Jin F Li B. The association of serum Klotho concentrations with hyperlipidemia prevalence and lipid levels among US adults: a cross-sectional study. BMC Public Health. (2023) 23:1645. 10.1186/s12889-023-16566-y 37641103 PMC10463308

[30] You Y Chen Y Fang W Li X Wang R Liu J. The association between sedentary behavior, exercise, and sleep disturbance: a mediation analysis of inflammatory biomarkers. Front Immunol. (2022) 13:1080782. 10.3389/fimmu.2022.1080782 36713451 PMC9880546

[31] Bakris G Ali W Parati G. ACC/AHA vs. ESC/ESH on hypertension guidelines: JACC guideline comparison. J Am Coll Cardiol. (2019) 73:3018–26. 10.1016/j.jacc.2019.03.507 31196460

[32] Cosentino F Grant PJ Aboyans V Bailey CJ Ceriello A Delgado V. 2019 ESC guidelines on diabetes, pre-diabetes, and cardiovascular diseases developed in collaboration with the EASD. Eur Heart J. (2020) 41:255–323. 10.1093/eurheartj/ehz486 31497854

[33] Kerr AG Andersson DP Ryden M Arner P. Insulin resistance in adipocytes: novel insights into the pathophysiology of metabolic syndrome. Clin Nutr. (2024) 43:468–75. 10.1016/j.clnu.2023.12.012 38181524

[34] Tang Y-M Chen X-G Jiao S-R. Advances of the study on the insulin resistance induced by high-fat diet. J Xihua Univ. 32:98–102.

[35] Isnanto R Setiawan I Gernowo R Warsito B Hadiyanto Warsito B. A systematic literature review on missing values: research trends, datasets, methods and frameworks. E3S Web Conf. (2023) 448:2020. 10.1051/e3sconf/202344802020

[36] Radosavljevic L Smith SM Nichols TE A. generative model for evaluating missing data methods in large epidemiological cohorts. BMC Med Res Methodol. (2025) 25:34. 10.1186/s12874-025-02487-4 39923001 PMC11806830

[37] Rezaie L Maazinezhad S Fogelberg DJ Khazaie H Sadeghi-Bahmani D Brand S. Compared to individuals with mild to moderate obstructive sleep apnea (OSA), individuals with severe OSA had higher BMI and respiratory-disturbance scores. Life. (2021) 11:368. 10.3390/life11050368 33919250 PMC8143081

[38] Zamarrón C Valdés Cuadrado L Alvarez-Sala R. Pathophysiologic mechanisms of cardiovascular disease in obstructive sleep apnea syndrome. Pulm Med. (2013) 2013:521087. 10.1155/2013/521087 23936649 PMC3712227

[39] Meng X Wen H Lian L. Association between triglyceride glucose-body mass index and obstructive sleep apnea: a study from NHANES 2015-2018. Front Nutr. (2024) 11:1424881. 10.3389/fnut.2024.1424881 39221158 PMC11363548

[40] Kim NH Park Y Kim NH Kim SG. Weight-adjusted waist index reflects fat and muscle mass in the opposite direction in older adults. Age Ageing. (2021) 50:780–6. 10.1093/ageing/afaa208 33035293

[41] Liu D Li Y Ji N Xia W Zhang B Feng X. Association between weight-adjusted waist index and testosterone deficiency in adult American men: findings from the national health and nutrition examination survey 2013-2016. BMC Public Health. (2024) 24:1683. 10.1186/s12889-024-19202-5 38915014 PMC11197353

[42] Kim JE Choi J Kim M Won CW. Assessment of existing anthropometric indices for screening sarcopenic obesity in older adults. Br J Nutr. (2023) 129:875–87. 10.1017/S0007114522001817 35710118 PMC9975784

[43] Li Q Qie R Qin P Zhang D Guo C Zhou Q. Association of weight-adjusted-waist index with incident hypertension: the rural Chinese cohort study. Nutr Metab Cardiovasc Dis. (2020) 30:1732–41. 10.1016/j.numecd.2020.05.033 32624344

[44] Kim JY Choi J Vella CA Criqui MH Allison MA Kim NH. Associations between weight-adjusted waist index and abdominal fat and muscle mass: multi-ethnic study of atherosclerosis. Diabetes Metab J. (2022) 46:747–55. 10.4093/dmj.2021.0294 35350091 PMC9532169

[45] Ong CW O'Driscoll DM Truby H Naughton MT Hamilton GS. The reciprocal interaction between obesity and obstructive sleep apnoea. Sleep Med Rev. (2013) 17:123–31. 10.1016/j.smrv.2012.05.002 22818968

[46] Schwartz AR Patil SP Laffan AM Polotsky V Schneider H Smith PL. Obesity and obstructive sleep apnea: pathogenic mechanisms and therapeutic approaches. Proc Am Thorac Soc. (2008) 5:185–92. 10.1513/pats.200708-137MG 18250211 PMC2645252

[47] Nokes B Orr JE White S Luu S Chen Z Alex R. Effect of obesity on sleep apnea pathogenesis differs in women vs. men: multiple mediation analyses in the retrospective SNOOzzzE cohort. J Appl Physiol. (2024) 136:1516–25. 10.1152/japplphysiol.00925.2023 38660729 PMC11368527

[48] Tao Z Zuo P Ma G. Association of weight-adjusted waist index with cardiovascular disease and mortality among metabolic syndrome population. Sci Rep. (2024) 14:18684. 10.1038/s41598-024-69486-1 39134613 PMC11319818

[49] Torén K Brisman J Järvholm B. Asthma and asthma-like symptoms in adults assessed by questionnaires. A literature review. Chest. (1993) 104:600–8. 10.1378/chest.104.2.600 7802735

[50] Leikauf J Federman AD. Comparisons of self-reported and chart-identified chronic diseases in inner-city seniors. J Am Geriatr Soc. (2009) 57:1219–25. 10.1111/j.1532-5415.2009.02313.x 19486197 PMC2768322

