# Supplementary material for: Non-linear association between weight-adjusted-waist index and obstructive sleep apnea: a cross-sectional study from the NHANES (2005–2008 to 2015–2020)
Source: Front Public Health. 2025 Mar 25;13:1546597. doi: 10.3389/fpubh.2025.1546597 (PMC11975944; doi:10.3389/fpubh.2025.1546597)
Supplement: Supplementary file 1 [file Table_1.docx]

**Supplemental table1**. Comparison of excluded and included characteristics

| WWI | Total (n = 12232) | Included(n = 11545) | excluded(n = 687) | Statistic | *P* |
| --- | --- | --- | --- | --- | --- |
|  |  |  |  |  |  |
| WC(cm) | 99.396 ± 16.627 | 99.391 ± 16.620 | 102.894 ± 21.149 | t=-0.868 | 0.385 |
| Weight(kg) | 81.988 ± 22.169 | 81.859 ± 21.515 | 85.008 ± 34.032 | t=-2.031 | 0.043 |
| Osa, n(%) |  |  |  | χ²=0.097 | **0.755** |
| No | 6159 (50.360) | 5818 (94.463) | 341 (5.537) |  |  |
| Yes | 6071 (49.640) | 5727 (94.334) | 344 (5.666) |  |  |
| Sex, n(%) |  |  |  | χ²=8.635 | 0.003 |
| Male | 5954 (48.676) | 5657 (95.012) | 297 (4.988) |  |  |
| Female | 6278 (51.324) | 5888 (93.788) | 390 (6.212) |  |  |
| Age, n(%) |  |  |  | χ²=70.440 | <.001 |
| <44 | 4957 (40.525) | 4737 (95.562) | 220 (4.438) |  |  |
| [44,60) | 3079 (25.172) | 2949 (95.778) | 130 (4.222) |  |  |
| ≥60 | 4196 (34.303) | 3859 (91.969) | 337 (8.031) |  |  |
| Race, n(%) |  |  |  | χ²=5.198 | 0.268 |
| Non-Hispanic Black | 1924 (15.729) | 1826 (94.906) | 98 (5.094) |  |  |
| Non-Hispanic White | 1215 (9.933) | 1148 (94.486) | 67 (5.514) |  |  |
| Other Hispanic | 4817 (39.380) | 4553 (94.519) | 264 (5.481) |  |  |
| Other Hispanic | 2877 (23.520) | 2692 (93.570) | 185 (6.430) |  |  |
| Other Races | 1399 (11.437) | 1326 (94.782) | 73 (5.218) |  |  |
| Educational level, n(%) |  |  |  | χ²=32.399 | <.001 |
| <High school | 2931 (24.618) | 2707 (92.358) | 224 (7.642) |  |  |
| High school | 2837 (23.828) | 2682 (94.536) | 155 (5.464) |  |  |
| >High school | 6138 (51.554) | 5849 (95.292) | 289 (4.708) |  |  |
| Marital status, n(%) |  |  |  | χ²=46.544 | <.001 |
| Married/Living with Partner | 7130 (59.871) | 6799 (95.358) | 331 (4.642) |  |  |
| Widowed/Divorced/Separated | 2682 (22.521) | 2462 (91.797) | 220 (8.203) |  |  |
| Never married | 2097 (17.609) | 1978 (94.325) | 119 (5.675) |  |  |
| PIR, n(%) |  |  |  | χ²=30.494 | <.001 |
| <1 | 2221 (20.276) | 2079 (93.606) | 142 (6.394) |  |  |
| [1, 3) | 4736 (43.235) | 4463 (94.236) | 273 (5.764) |  |  |
| ≥ 3 | 3997 (36.489) | 3853 (96.397) | 144 (3.603) |  |  |
| BMI, n(%) |  |  |  | χ²=2.136 | 0.344 |
| ≤25 | 3528 (29.373) | 3390 (96.088) | 138 (3.912) |  |  |
| (25,30] | 3928 (32.703) | 3783 (96.309) | 145 (3.691) |  |  |
| >30 | 4555 (37.924) | 4359 (95.697) | 196 (4.303) |  |  |
| Alcohol consumption, n(%) |  |  |  | χ²=64.221 | <.001 |
| Never | 2262 (23.167) | 2132 (94.253) | 130 (5.747) |  |  |
| Moderate | 6369 (65.229) | 6223 (97.708) | 146 (2.292) |  |  |
| Heavy | 1133 (11.604) | 1096 (96.734) | 37 (3.266) |  |  |
| Smoking, n(%) |  |  |  | χ²=5.008 | 0.082 |
| Never | 6805 (55.701) | 6402 (94.078) | 403 (5.922) |  |  |
| Former | 2950 (24.147) | 2784 (94.373) | 166 (5.627) |  |  |
| Now | 2462 (20.152) | 2346 (95.288) | 116 (4.712) |  |  |
| HBP, n(%) |  |  |  | χ²=24.231 | <.001 |
| No | 7093 (57.992) | 6757 (95.263) | 336 (4.737) |  |  |
| Yes | 5138 (42.008) | 4788 (93.188) | 350 (6.812) |  |  |
| Diabetes, n(%) |  |  |  | χ²=35.842 | <.001 |
| No | 9913 (81.042) | 9416 (94.986) | 497 (5.014) |  |  |
| Yes | 2319 (18.958) | 2129 (91.807) | 190 (8.193) |  |  |
| CHD, n(%) |  |  |  | χ²=23.726 | <.001 |
| No | 11342 (95.624) | 10732 (94.622) | 610 (5.378) |  |  |
| Yes | 519 (4.376) | 465 (89.595) | 54 (10.405) |  |  |
| Sleep duration, n(%) |  |  |  | χ²=20.444 | <.001 |
| <7 | 3710 (30.455) | 3509 (94.582) | 201 (5.418) |  |  |
| [7, 9) | 6371 (52.298) | 6048 (94.930) | 323 (5.070) |  |  |
| ≥9 | 2101 (17.247) | 1940 (92.337) | 161 (7.663) |  |  |
| Osa, n(%) |  |  |  | χ²=0.097 | 0.755 |
| No | 6159 (50.360) | 5818 (94.463) | 341 (5.537) |  |  |
| Yes | 6071 (49.640) | 5727 (94.334) | 344 (5.666) |  |  |
| t: t-test, χ²: Chi-square test |  |  |  |  |  |
| SD: standard deviation |  |  |  |  |  |

| WWI | Total (n = 12232) | Included(n = 11545) | excluded(n = 687) | Statistic | P |
| --- | --- | --- | --- | --- | --- |
| WC(cm) | 99.40 ± 16.63 | 99.39 ± 16.62 | 102.89 ± 21.15 | t=-0.87 | 0.385 |
| Weight(kg) | 81.99 ± 22.17 | 81.86 ± 21.52 | 85.01 ± 34.03 | t=-2.03 | 0.043 |
| Osa, n(%) |  |  |  | χ²=0.10 | 0.755 |
| No | 6159 (50.36) | 5818 (94.46) | 341 (5.54) |  |  |
| Yes | 6071 (49.64) | 5727 (94.33) | 344 (5.67) |  |  |
| Sex, n(%) |  |  |  | χ²=8.64 | 0.003 |
| Male | 5954 (48.68) | 5657 (95.01) | 297 (4.99) |  |  |
| Female | 6278 (51.32) | 5888 (93.79) | 390 (6.21) |  |  |
| Age, n(%) |  |  |  | χ²=70.44 | <.001 |
| <44 | 4957 (40.53) | 4737 (95.56) | 220 (4.44) |  |  |
| [44,60) | 3079 (25.17) | 2949 (95.78) | 130 (4.22) |  |  |
| ≥60 | 4196 (34.30) | 3859 (91.97) | 337 (8.03) |  |  |
| Race, n(%) |  |  |  | χ²=5.20 | 0.268 |
| Non-Hispanic Black | 1924 (15.73) | 1826 (94.91) | 98 (5.09) |  |  |
| Non-Hispanic White | 1215 (9.93) | 1148 (94.49) | 67 (5.51) |  |  |
| Other Hispanic | 4817 (39.38) | 4553 (94.52) | 264 (5.48) |  |  |
| Other Hispanic | 2877 (23.52) | 2692 (93.57) | 185 (6.43) |  |  |
| Other Races | 1399 (11.44) | 1326 (94.78) | 73 (5.22) |  |  |
| Educational level, n(%) |  |  |  | χ²=32.40 | <.001 |
| <High school | 2931 (24.62) | 2707 (92.36) | 224 (7.64) |  |  |
| High school | 2837 (23.83) | 2682 (94.54) | 155 (5.46) |  |  |
| >High school | 6138 (51.55) | 5849 (95.29) | 289 (4.71) |  |  |
| Marital status, n(%) |  |  |  | χ²=46.54 | <.001 |
| Married/Living with Partner | 7130 (59.87) | 6799 (95.36) | 331 (4.64) |  |  |
| Widowed/Divorced/Separated | 2682 (22.52) | 2462 (91.80) | 220 (8.20) |  |  |
| Never married | 2097 (17.61) | 1978 (94.31) | 119 (5.68) |  |  |
| PIR, n(%) |  |  |  | χ²=30.49 | <.001 |
| <1 | 2221 (20.28) | 2079 (93.61) | 142 (6.39) |  |  |
| [1, 3) | 4736 (43.24) | 4463 (94.24) | 273 (5.76) |  |  |
| ≥ 3 | 3997 (36.49) | 3853 (96.40) | 144 (3.60) |  |  |
| BMI, n(%) |  |  |  | χ²=2.14 | 0.344 |
| ≤25 | 3528 (29.37) | 3390 (96.09) | 138 (3.91) |  |  |
| (25,30] | 3928 (32.70) | 3783 (96.31) | 145 (3.69) |  |  |
| >30 | 4555 (37.92) | 4359 (95.70) | 196 (4.30) |  |  |
| Alcohol consumption, n(%) |  |  |  | χ²=64.22 | <.001 |
| Never | 2262 (23.17) | 2132 (94.25) | 130 (5.75) |  |  |
| Moderate | 6369 (65.23) | 6223 (97.71) | 146 (2.29) |  |  |
| Heavy | 1133 (11.60) | 1096 (96.73) | 37 (3.267) |  |  |
| Smoking, n(%) |  |  |  | χ²=5.01 | 0.082 |
| Never | 6805 (55.70) | 6402 (94.08) | 403 (5.92) |  |  |
| Former | 2950 (24.15) | 2784 (94.37) | 166 (5.63) |  |  |
| Now | 2462 (20.15) | 2346 (95.29) | 116 (4.71) |  |  |
| HBP, n(%) |  |  |  | χ²=24.23 | <.001 |
| No | 7093 (57.99) | 6757 (95.26 | 336 (4.74 |  |  |
| Yes | 5138 (42.01) | 4788 (93.19) | 350 (6.81) |  |  |
| Diabetes, n(%) |  |  |  | χ²=35.84 | <.001 |
| No | 9913 (81.04) | 9416 (94.97) | 497 (5.01) |  |  |
| Yes | 2319 (18.96) | 2129 (91.81) | 190 (8.19) |  |  |
| CHD, n(%) |  |  |  | χ²=23.73 | <.001 |
| No | 11342 (95.62) | 10732 (94.62) | 610 (5.38) |  |  |
| Yes | 519 (4.38) | 465 (89.60) | 54 (10.41) |  |  |
| Sleep duration, n(%) |  |  |  | χ²=20.44 | <.001 |
| <7 | 3710 (30.46) | 3509 (94.58) | 201 (5.42) |  |  |
| [7, 9) | 6371 (52.30) | 6048 (94.93) | 323 (5.07) |  |  |
| ≥9 | 2101 (17.25) | 1940 (92.34) | 161 (7.66) |  |  |
| Osa, n(%) |  |  |  | χ²=0.10 | 0.755 |
| No | 6159 (50.36) | 5818 (94.46) | 341 (5.54) |  |  |
| Yes | 6071 (49.64) | 5727 (94.33) | 344 (5.67) |  |  |
| t: t-test, χ²: Chi-square test |  |  |  |  |  |
| SD: standard deviation |  |  |  |  |  |
|  |  |  |  |  |  |
