# Supplementary material for: Non-linear association between weight-adjusted-waist index and obstructive sleep apnea: a cross-sectional study from the NHANES (2005–2008 to 2015–2020)
Source: Front Public Health. 2025 Mar 25;13:1546597. doi: 10.3389/fpubh.2025.1546597 (PMC11975944; doi:10.3389/fpubh.2025.1546597)
Supplement: Supplementary file 2 [file Data_Sheet_1.zip › Raw/Figure3/age/20052020_20_tbl/20052020_20_tbl.htm]

## 单因素分析

Outcome: OSA
Exposure: WWI
Adjust for: SEX RACE EDUCATIONAL\_LEVEL MARITAL\_STATUS ALCOHOL\_CONSUMPTION SMOKING HBP DIABETES CHD SLEEP\_DURATION PIR
svy.DSN<-svydesign(id=~SDMVPS\_U, strata=~SDMVSTR\_A,weights=~WTSAF2Y\_R, data=WD,nest=TRUE)

|  |  |  |  |  |  |  |  |
| --- | --- | --- | --- | --- | --- | --- | --- |
|  | AGE= 1 | AGE= 1 | AGE= 2 | AGE= 2 | AGE= 3 | AGE= 3 | P-interaction |
| Outcome: OSA | (N) % (95%CI) | OR (95%CI) P-value | (N) % (95%CI) | OR (95%CI) P-value | (N) % (95%CI) | OR (95%CI) P-value |  |
| WWI | (4150) 42.886 (40.748 ,45.025) | 1.737 (1.564, 1.930) <0.0001 | (2662) 58.216 (55.648 ,60.784) | 1.560 (1.306, 1.863) <0.0001 | (3433) 49.339 (46.928 ,51.751) | 1.296 (1.125, 1.494) 0.0009 | 0.0057 |

Data in table:
N: Number of observed
 % (95%CI): survey-weighted percentage (95% CI)
For
OSA
: survey-weighted OR (95%CI) p-value
P-interaction: by global Chi-square test for interaction terms (exposure:
AGE
)
Created by EmpowerStats (www.empowerstats.com) and R on 2024-10-14
