# Supplementary material for: Non-linear association between weight-adjusted-waist index and obstructive sleep apnea: a cross-sectional study from the NHANES (2005–2008 to 2015–2020)
Source: Front Public Health. 2025 Mar 25;13:1546597. doi: 10.3389/fpubh.2025.1546597 (PMC11975944; doi:10.3389/fpubh.2025.1546597)
Supplement: Supplementary file 2 [file Data_Sheet_1.zip › Raw/Figure3/alcohol consumption/20052020_25_tbl/20052020_25_tbl.htm]

## 单因素分析

Outcome: OSA
Exposure: WWI
Adjust for: SEX AGE EDUCATIONAL\_LEVEL RACE PIR SMOKING HBP DIABETES CHD SLEEP\_DURATION MARITAL\_STATUS
svy.DSN<-svydesign(id=~SDMVPS\_U, strata=~SDMVSTR\_A,weights=~WTSAF2Y\_R, data=WD,nest=TRUE)

|  |  |  |  |  |  |  |  |  |  |
| --- | --- | --- | --- | --- | --- | --- | --- | --- | --- |
|  | ALCOHOL\_CONSUMPTION= 0 | ALCOHOL\_CONSUMPTION= 0 | ALCOHOL\_CONSUMPTION= 1 | ALCOHOL\_CONSUMPTION= 1 | ALCOHOL\_CONSUMPTION= 2 | ALCOHOL\_CONSUMPTION= 2 | ALCOHOL\_CONSUMPTION= 9 | ALCOHOL\_CONSUMPTION= 9 | P-interaction |
| Outcome: OSA | (N) % (95%CI) | OR (95%CI) P-value | (N) % (95%CI) | OR (95%CI) P-value | (N) % (95%CI) | OR (95%CI) P-value | (N) % (95%CI) | OR (95%CI) P-value |  |
| WWI | (1903) 53.710 (50.740 ,56.680) | 1.507 (1.255, 1.809) 0.0001 | (5594) 49.643 (47.654 ,51.633) | 1.556 (1.411, 1.715) <0.0001 | (986) 48.928 (43.346 ,54.510) | 2.044 (1.579, 2.646) <0.0001 | (1762) 42.070 (39.197 ,44.943) | 1.513 (1.276, 1.794) <0.0001 | 0.2127 |

Data in table:
N: Number of observed
 % (95%CI): survey-weighted percentage (95% CI)
For
OSA
: survey-weighted OR (95%CI) p-value
P-interaction: by global Chi-square test for interaction terms (exposure:
ALCOHOL\_CONSUMPTION
)
Created by EmpowerStats (www.empowerstats.com) and R on 2024-10-14
