# Supplementary material for: Non-linear association between weight-adjusted-waist index and obstructive sleep apnea: a cross-sectional study from the NHANES (2005–2008 to 2015–2020)
Source: Front Public Health. 2025 Mar 25;13:1546597. doi: 10.3389/fpubh.2025.1546597 (PMC11975944; doi:10.3389/fpubh.2025.1546597)
Supplement: Supplementary file 2 [file Data_Sheet_1.zip › Raw/Figure3/coronary heart disease/20052020_29_tbl/20052020_29_tbl.htm]

## 单因素分析

Outcome: OSA
Exposure: WWI
Adjust for: SEX AGE EDUCATIONAL\_LEVEL RACE PIR ALCOHOL\_CONSUMPTION SMOKING HBP DIABETES SLEEP\_DURATION MARITAL\_STATUS
svy.DSN<-svydesign(id=~SDMVPS\_U, strata=~SDMVSTR\_A,weights=~WTSAF2Y\_R, data=WD,nest=TRUE)

|  |  |  |  |  |  |  |  |
| --- | --- | --- | --- | --- | --- | --- | --- |
|  | CHD= 0 | CHD= 0 | CHD= 1 | CHD= 1 | CHD= 9 | CHD= 9 | P-interaction |
| Outcome: OSA | (N) % (95%CI) | OR (95%CI) P-value | (N) % (95%CI) | OR (95%CI) P-value | (N) % (95%CI) | OR (95%CI) P-value |  |
| WWI | (9540) 49.194 (47.544 ,50.844) | 1.566 (1.437, 1.706) <0.0001 | (414) 55.066 (48.226 ,61.906) | 1.487 (1.074, 2.058) 0.0217 | (291) 33.176 (25.386 ,40.966) | 2.058 (1.388, 3.052) 0.0009 | 0.3496 |

Data in table:
N: Number of observed
 % (95%CI): survey-weighted percentage (95% CI)
For
OSA
: survey-weighted OR (95%CI) p-value
P-interaction: by global Chi-square test for interaction terms (exposure:
CHD
)
Created by EmpowerStats (www.empowerstats.com) and R on 2024-10-14
