# Supplementary material for: Non-linear association between weight-adjusted-waist index and obstructive sleep apnea: a cross-sectional study from the NHANES (2005–2008 to 2015–2020)
Source: Front Public Health. 2025 Mar 25;13:1546597. doi: 10.3389/fpubh.2025.1546597 (PMC11975944; doi:10.3389/fpubh.2025.1546597)
Supplement: Supplementary file 2 [file Data_Sheet_1.zip › Raw/Figure3/diabetes/20052020_28_tbl/20052020_28_tbl.htm]

## 单因素分析

Outcome: OSA
Exposure: WWI
Adjust for: SEX AGE EDUCATIONAL\_LEVEL RACE PIR ALCOHOL\_CONSUMPTION SMOKING HBP CHD SLEEP\_DURATION MARITAL\_STATUS
svy.DSN<-svydesign(id=~SDMVPS\_U, strata=~SDMVSTR\_A,weights=~WTSAF2Y\_R, data=WD,nest=TRUE)

|  |  |  |  |  |  |
| --- | --- | --- | --- | --- | --- |
|  | DIABETES= 0 | DIABETES= 0 | DIABETES= 1 | DIABETES= 1 | P-interaction |
| Outcome: OSA | (N) % (95%CI) | OR (95%CI) P-value | (N) % (95%CI) | OR (95%CI) P-value |  |
| WWI | (8333) 47.439 (45.688 ,49.190) | 1.571 (1.447, 1.705) <0.0001 | (1912) 60.053 (56.850 ,63.256) | 1.585 (1.286, 1.952) 0.0001 | 0.9253 |

Data in table:
N: Number of observed
 % (95%CI): survey-weighted percentage (95% CI)
For
OSA
: survey-weighted OR (95%CI) p-value
P-interaction: by global Chi-square test for interaction terms (exposure:
DIABETES
)
Created by EmpowerStats (www.empowerstats.com) and R on 2024-10-14
