# Supplementary material for: Non-linear association between weight-adjusted-waist index and obstructive sleep apnea: a cross-sectional study from the NHANES (2005–2008 to 2015–2020)
Source: Front Public Health. 2025 Mar 25;13:1546597. doi: 10.3389/fpubh.2025.1546597 (PMC11975944; doi:10.3389/fpubh.2025.1546597)
Supplement: Supplementary file 2 [file Data_Sheet_1.zip › Raw/Figure3/education level/20052020_22_tbl/20052020_22_tbl.htm]

## 单因素分析

Outcome: OSA
Exposure: WWI
Adjust for: SEX AGE RACE MARITAL\_STATUS ALCOHOL\_CONSUMPTION SMOKING HBP DIABETES CHD SLEEP\_DURATION PIR
svy.DSN<-svydesign(id=~SDMVPS\_U, strata=~SDMVSTR\_A,weights=~WTSAF2Y\_R, data=WD,nest=TRUE)

|  |  |  |  |  |  |  |  |  |  |
| --- | --- | --- | --- | --- | --- | --- | --- | --- | --- |
|  | EDUCATIONAL\_LEVEL= 1 | EDUCATIONAL\_LEVEL= 1 | EDUCATIONAL\_LEVEL= 2 | EDUCATIONAL\_LEVEL= 2 | EDUCATIONAL\_LEVEL= 3 | EDUCATIONAL\_LEVEL= 3 | EDUCATIONAL\_LEVEL= 9 | EDUCATIONAL\_LEVEL= 9 | P-interaction |
| Outcome: OSA | (N) % (95%CI) | OR (95%CI) P-value | (N) % (95%CI) | OR (95%CI) P-value | (N) % (95%CI) | OR (95%CI) P-value | (N) % (95%CI) | OR (95%CI) P-value |  |
| WWI | (2347) 53.907 (50.587 ,57.227) | 1.529 (1.333, 1.755) <0.0001 | (2379) 53.252 (50.144 ,56.361) | 1.368 (1.166, 1.604) 0.0004 | (5263) 46.744 (44.802 ,48.685) | 1.660 (1.499, 1.838) <0.0001 | (256) 27.773 (20.183 ,35.363) | 2.242 (1.560, 3.222) 0.0001 | 0.0178 |

Data in table:
N: Number of observed
 % (95%CI): survey-weighted percentage (95% CI)
For
OSA
: survey-weighted OR (95%CI) p-value
P-interaction: by global Chi-square test for interaction terms (exposure:
EDUCATIONAL\_LEVEL
)
Created by EmpowerStats (www.empowerstats.com) and R on 2024-10-14
