# Supplementary material for: Non-linear association between weight-adjusted-waist index and obstructive sleep apnea: a cross-sectional study from the NHANES (2005–2008 to 2015–2020)
Source: Front Public Health. 2025 Mar 25;13:1546597. doi: 10.3389/fpubh.2025.1546597 (PMC11975944; doi:10.3389/fpubh.2025.1546597)
Supplement: Supplementary file 2 [file Data_Sheet_1.zip › Raw/Figure3/marital status/20052020_23_tbl/20052020_23_tbl.htm]

## 单因素分析

Outcome: OSA
Exposure: WWI
Adjust for: SEX AGE EDUCATIONAL\_LEVEL RACE ALCOHOL\_CONSUMPTION SMOKING HBP DIABETES CHD SLEEP\_DURATION PIR
svy.DSN<-svydesign(id=~SDMVPS\_U, strata=~SDMVSTR\_A,weights=~WTSAF2Y\_R, data=WD,nest=TRUE)

|  |  |  |  |  |  |  |  |  |  |
| --- | --- | --- | --- | --- | --- | --- | --- | --- | --- |
|  | MARITAL\_STATUS= 1 | MARITAL\_STATUS= 1 | MARITAL\_STATUS= 2 | MARITAL\_STATUS= 2 | MARITAL\_STATUS= 3 | MARITAL\_STATUS= 3 | MARITAL\_STATUS= 9 | MARITAL\_STATUS= 9 | P-interaction |
| Outcome: OSA | (N) % (95%CI) | OR (95%CI) P-value | (N) % (95%CI) | OR (95%CI) P-value | (N) % (95%CI) | OR (95%CI) P-value | (N) % (95%CI) | OR (95%CI) P-value |  |
| WWI | (6120) 53.119 (51.356 ,54.882) | 1.601 (1.448, 1.770) <0.0001 | (2142) 46.055 (42.754 ,49.356) | 1.474 (1.261, 1.724) <0.0001 | (1728) 39.326 (36.045 ,42.606) | 1.530 (1.299, 1.803) <0.0001 | (255) 28.037 (20.467 ,35.607) | 2.229 (1.551, 3.203) 0.0001 | 0.0955 |

Data in table:
N: Number of observed
 % (95%CI): survey-weighted percentage (95% CI)
For
OSA
: survey-weighted OR (95%CI) p-value
P-interaction: by global Chi-square test for interaction terms (exposure:
MARITAL\_STATUS
)
Created by EmpowerStats (www.empowerstats.com) and R on 2024-10-14
