# Supplementary material for: Non-linear association between weight-adjusted-waist index and obstructive sleep apnea: a cross-sectional study from the NHANES (2005–2008 to 2015–2020)
Source: Front Public Health. 2025 Mar 25;13:1546597. doi: 10.3389/fpubh.2025.1546597 (PMC11975944; doi:10.3389/fpubh.2025.1546597)
Supplement: Supplementary file 2 [file Data_Sheet_1.zip › Raw/Figure3/race/20052020_21_tbl/20052020_21_tbl.htm]

## 单因素分析

Outcome: OSA
Exposure: WWI
Adjust for: SEX AGE EDUCATIONAL\_LEVEL MARITAL\_STATUS ALCOHOL\_CONSUMPTION SMOKING HBP DIABETES CHD SLEEP\_DURATION PIR
svy.DSN<-svydesign(id=~SDMVPS\_U, strata=~SDMVSTR\_A,weights=~WTSAF2Y\_R, data=WD,nest=TRUE)

|  |  |  |  |  |  |  |  |  |  |  |  |
| --- | --- | --- | --- | --- | --- | --- | --- | --- | --- | --- | --- |
|  | RACE= 1 | RACE= 1 | RACE= 2 | RACE= 2 | RACE= 3 | RACE= 3 | RACE= 4 | RACE= 4 | RACE= 5 | RACE= 5 | P-interaction |
| Outcome: OSA | (N) % (95%CI) | OR (95%CI) P-value | (N) % (95%CI) | OR (95%CI) P-value | (N) % (95%CI) | OR (95%CI) P-value | (N) % (95%CI) | OR (95%CI) P-value | (N) % (95%CI) | OR (95%CI) P-value |  |
| WWI | (1670) 49.711 (46.820 ,52.601) | 1.652 (1.438, 1.898) <0.0001 | (1040) 49.541 (45.376 ,53.705) | 1.514 (1.269, 1.808) <0.0001 | (4097) 48.861 (46.677 ,51.045) | 1.569 (1.411, 1.744) <0.0001 | (2241) 50.437 (48.243 ,52.632) | 1.442 (1.271, 1.637) <0.0001 | (1197) 48.362 (43.757 ,52.968) | 1.856 (1.513, 2.278) <0.0001 | 0.1473 |

Data in table:
N: Number of observed
 % (95%CI): survey-weighted percentage (95% CI)
For
OSA
: survey-weighted OR (95%CI) p-value
P-interaction: by global Chi-square test for interaction terms (exposure:
RACE
)
Created by EmpowerStats (www.empowerstats.com) and R on 2024-10-14
