# Supplementary material for: Non-linear association between weight-adjusted-waist index and obstructive sleep apnea: a cross-sectional study from the NHANES (2005–2008 to 2015–2020)
Source: Front Public Health. 2025 Mar 25;13:1546597. doi: 10.3389/fpubh.2025.1546597 (PMC11975944; doi:10.3389/fpubh.2025.1546597)
Supplement: Supplementary file 2 [file Data_Sheet_1.zip › Raw/Figure3/ratio of family income to poverty/20052020_24_tbl/20052020_24_tbl.htm]

## 单因素分析

Outcome: OSA
Exposure: WWI
Adjust for: SEX AGE EDUCATIONAL\_LEVEL RACE ALCOHOL\_CONSUMPTION SMOKING HBP DIABETES CHD SLEEP\_DURATION MARITAL\_STATUS
svy.DSN<-svydesign(id=~SDMVPS\_U, strata=~SDMVSTR\_A,weights=~WTSAF2Y\_R, data=WD,nest=TRUE)

|  |  |  |  |  |  |  |  |  |  |
| --- | --- | --- | --- | --- | --- | --- | --- | --- | --- |
|  | PIR= 1 | PIR= 1 | PIR= 2 | PIR= 2 | PIR= 3 | PIR= 3 | PIR= 9 | PIR= 9 | P-interaction |
| Outcome: OSA | (N) % (95%CI) | OR (95%CI) P-value | (N) % (95%CI) | OR (95%CI) P-value | (N) % (95%CI) | OR (95%CI) P-value | (N) % (95%CI) | OR (95%CI) P-value |  |
| WWI | (1802) 50.324 (47.127 ,53.520) | 1.421 (1.261, 1.601) <0.0001 | (3963) 50.169 (47.839 ,52.499) | 1.442 (1.293, 1.609) <0.0001 | (3504) 48.605 (46.216 ,50.994) | 1.738 (1.523, 1.984) <0.0001 | (976) 45.393 (41.741 ,49.044) | 1.608 (1.306, 1.980) 0.0001 | 0.0562 |

Data in table:
N: Number of observed
 % (95%CI): survey-weighted percentage (95% CI)
For
OSA
: survey-weighted OR (95%CI) p-value
P-interaction: by global Chi-square test for interaction terms (exposure:
PIR
)
Created by EmpowerStats (www.empowerstats.com) and R on 2024-10-14
