# Supplementary material for: Non-linear association between weight-adjusted-waist index and obstructive sleep apnea: a cross-sectional study from the NHANES (2005–2008 to 2015–2020)
Source: Front Public Health. 2025 Mar 25;13:1546597. doi: 10.3389/fpubh.2025.1546597 (PMC11975944; doi:10.3389/fpubh.2025.1546597)
Supplement: Supplementary file 2 [file Data_Sheet_1.zip › Raw/Figure3/sex/20052020_19_tbl/20052020_19_tbl.htm]

## 单因素分析

Outcome: OSA
Exposure: WWI
Adjust for: AGE RACE EDUCATIONAL\_LEVEL MARITAL\_STATUS ALCOHOL\_CONSUMPTION SMOKING HBP DIABETES CHD SLEEP\_DURATION PIR
svy.DSN<-svydesign(id=~SDMVPS\_U, strata=~SDMVSTR\_A,weights=~WTSAF2Y\_R, data=WD,nest=TRUE)

|  |  |  |  |  |  |
| --- | --- | --- | --- | --- | --- |
|  | SEX= 1 | SEX= 1 | SEX= 2 | SEX= 2 | P-interaction |
| Outcome: OSA | (N) % (95%CI) | OR (95%CI) P-value | (N) % (95%CI) | OR (95%CI) P-value |  |
| WWI | (4989) 55.609 (53.684 ,57.534) | 1.595 (1.401, 1.814) <0.0001 | (5256) 42.975 (40.942 ,45.009) | 1.556 (1.416, 1.710) <0.0001 | 0.7356 |

Data in table:
N: Number of observed
 % (95%CI): survey-weighted percentage (95% CI)
For
OSA
: survey-weighted OR (95%CI) p-value
P-interaction: by global Chi-square test for interaction terms (exposure:
SEX
)
Created by EmpowerStats (www.empowerstats.com) and R on 2024-10-14
