# Supplementary material for: Non-linear association between weight-adjusted-waist index and obstructive sleep apnea: a cross-sectional study from the NHANES (2005–2008 to 2015–2020)
Source: Front Public Health. 2025 Mar 25;13:1546597. doi: 10.3389/fpubh.2025.1546597 (PMC11975944; doi:10.3389/fpubh.2025.1546597)
Supplement: Supplementary file 2 [file Data_Sheet_1.zip › Raw/Figure3/sleep duration/20052020_30_tbl/20052020_30_tbl.htm]

## 单因素分析

Outcome: OSA
Exposure: WWI
Adjust for: SEX AGE EDUCATIONAL\_LEVEL RACE PIR ALCOHOL\_CONSUMPTION SMOKING HBP DIABETES MARITAL\_STATUS CHD
svy.DSN<-svydesign(id=~SDMVPS\_U, strata=~SDMVSTR\_A,weights=~WTSAF2Y\_R, data=WD,nest=TRUE)

|  |  |  |  |  |  |  |  |  |  |
| --- | --- | --- | --- | --- | --- | --- | --- | --- | --- |
|  | SLEEP\_DURATION= 1 | SLEEP\_DURATION= 1 | SLEEP\_DURATION= 2 | SLEEP\_DURATION= 2 | SLEEP\_DURATION= 3 | SLEEP\_DURATION= 3 | SLEEP\_DURATION= 9 | SLEEP\_DURATION= 9 | P-interaction |
| Outcome: OSA | (N) % (95%CI) | OR (95%CI) P-value | (N) % (95%CI) | OR (95%CI) P-value | (N) % (95%CI) | OR (95%CI) P-value | (N) % (95%CI) | OR (95%CI) P-value |  |
| WWI | (3094) 55.702 (53.337 ,58.067) | 1.425 (1.240, 1.637) <0.0001 | (5421) 46.915 (44.936 ,48.893) | 1.658 (1.494, 1.840) <0.0001 | (1688) 44.591 (41.639 ,47.542) | 1.547 (1.339, 1.788) <0.0001 | (42) 49.933 (17.487 ,82.378) | 1.325 (0.372, 4.716) 0.6665 | 0.2693 |

Data in table:
N: Number of observed
 % (95%CI): survey-weighted percentage (95% CI)
For
OSA
: survey-weighted OR (95%CI) p-value
P-interaction: by global Chi-square test for interaction terms (exposure:
SLEEP\_DURATION
)
Created by EmpowerStats (www.empowerstats.com) and R on 2024-10-14
