# Supplementary material for: Non-linear association between weight-adjusted-waist index and obstructive sleep apnea: a cross-sectional study from the NHANES (2005–2008 to 2015–2020)
Source: Front Public Health. 2025 Mar 25;13:1546597. doi: 10.3389/fpubh.2025.1546597 (PMC11975944; doi:10.3389/fpubh.2025.1546597)
Supplement: Supplementary file 2 [file Data_Sheet_1.zip › Raw/Figure3/smoking/20052020_26_tbl/20052020_26_tbl.htm]

## 单因素分析

Outcome: OSA
Exposure: WWI
Adjust for: SEX AGE EDUCATIONAL\_LEVEL RACE PIR ALCOHOL\_CONSUMPTION HBP DIABETES CHD SLEEP\_DURATION MARITAL\_STATUS
svy.DSN<-svydesign(id=~SDMVPS\_U, strata=~SDMVSTR\_A,weights=~WTSAF2Y\_R, data=WD,nest=TRUE)

|  |  |  |  |  |  |  |  |  |  |
| --- | --- | --- | --- | --- | --- | --- | --- | --- | --- |
|  | SMOKING= 0 | SMOKING= 0 | SMOKING= 1 | SMOKING= 1 | SMOKING= 2 | SMOKING= 2 | SMOKING= 9 | SMOKING= 9 | P-interaction |
| Outcome: OSA | (N) % (95%CI) | OR (95%CI) P-value | (N) % (95%CI) | OR (95%CI) P-value | (N) % (95%CI) | OR (95%CI) P-value | (N) % (95%CI) | OR (95%CI) P-value |  |
| WWI | (5731) 44.711 (42.837 ,46.585) | 1.647 (1.481, 1.831) <0.0001 | (2521) 52.624 (50.155 ,55.093) | 1.595 (1.366, 1.862) <0.0001 | (1981) 56.581 (53.527 ,59.636) | 1.369 (1.181, 1.587) 0.0002 | (12) 39.028 (6.954 ,71.102) | 0.512 (0.145, 1.810) 0.3053 | 0.0195 |

Data in table:
N: Number of observed
 % (95%CI): survey-weighted percentage (95% CI)
For
OSA
: survey-weighted OR (95%CI) p-value
P-interaction: by global Chi-square test for interaction terms (exposure:
SMOKING
)
Created by EmpowerStats (www.empowerstats.com) and R on 2024-10-14
