# Supplementary material for: Non-linear association between weight-adjusted-waist index and obstructive sleep apnea: a cross-sectional study from the NHANES (2005–2008 to 2015–2020)
Source: Front Public Health. 2025 Mar 25;13:1546597. doi: 10.3389/fpubh.2025.1546597 (PMC11975944; doi:10.3389/fpubh.2025.1546597)
Supplement: Supplementary file 2 [file Data_Sheet_1.zip › Raw/table1/20052020_2_tbl/20052020_2_tbl.htm]

## 研究人群描述

|  |  |
| --- | --- |
|  |  |
|  | Mean+SD |
| WC | 99.391 ± 16.620 |
| WEIGHT | 81.859 ± 21.515 |
|  | N (%) |
| RACE |  |
| 1 | 1826 (15.816%) |
| 2 | 1148 (9.944%) |
| 3 | 4553 (39.437%) |
| 4 | 2692 (23.317%) |
| 5 | 1326 (11.485%) |
| SEX |  |
| 1 | 5657 (49.000%) |
| 2 | 5888 (51.000%) |
| AGE |  |
| 1 | 4737 (41.031%) |
| 2 | 2949 (25.544%) |
| 3 | 3859 (33.426%) |
| EDUCATIONAL\_LEVEL |  |
| 1 | 2707 (23.447%) |
| 2 | 2682 (23.231%) |
| 3 | 5849 (50.663%) |
| 9 | 307 (2.659%) |
| MARITAL\_STATUS |  |
| 1 | 6799 (58.891%) |
| 2 | 2462 (21.325%) |
| 3 | 1978 (17.133%) |
| 9 | 306 (2.650%) |
| PIR |  |
| 1 | 2079 (18.008%) |
| 2 | 4463 (38.657%) |
| 3 | 3853 (33.374%) |
| 9 | 1150 (9.961%) |
| BMI |  |
| 1 | 3390 (29.396%) |
| 2 | 3783 (32.804%) |
| 3 | 4359 (37.799%) |
| ALCOHOL\_CONSUMPTION |  |
| 0 | 2132 (18.467%) |
| 1 | 6223 (53.902%) |
| 2 | 1096 (9.493%) |
| 9 | 2094 (18.138%) |
| SMOKING |  |
| 0 | 6402 (55.453%) |
| 1 | 2784 (24.114%) |
| 2 | 2346 (20.320%) |
| 9 | 13 (0.113%) |
| HBP |  |
| 0 | 6757 (58.528%) |
| 1 | 4788 (41.472%) |
| DIABETES |  |
| 0 | 9416 (81.559%) |
| 1 | 2129 (18.441%) |
| CHD |  |
| 0 | 10732 (92.958%) |
| 1 | 465 (4.028%) |
| 9 | 348 (3.014%) |
| SLEEP\_DURATION |  |
| 1 | 3509 (30.394%) |
| 2 | 6048 (52.386%) |
| 3 | 1940 (16.804%) |
| 9 | 48 (0.416%) |
| OSA |  |
| 0 | 5818 (50.394%) |
| 1 | 5727 (49.606%) |

表中结果:
Mean+SD / N(%)
此表用易侕统计软件 (www.empowerstats.com) 和R软件生成，生成日期： 2024-10-13
