# Supplementary material for: Non-linear association between weight-adjusted-waist index and obstructive sleep apnea: a cross-sectional study from the NHANES (2005–2008 to 2015–2020)
Source: Front Public Health. 2025 Mar 25;13:1546597. doi: 10.3389/fpubh.2025.1546597 (PMC11975944; doi:10.3389/fpubh.2025.1546597)
Supplement: Supplementary file 2 [file Data_Sheet_1.zip › Raw/table1/20052020_3_tbl/20052020_3_tbl.htm]

## 研究人群描述

|  |  |  |  |  |  |  |
| --- | --- | --- | --- | --- | --- | --- |
| WWI quartile | Q1 | Q2 | Q3 | Q4 | P-value | P-value\* |
| N | 2886 | 2886 | 2886 | 2887 |  |  |
| WC | 84.588 ± 10.663 | 96.322 ± 11.839 | 103.437 ± 13.047 | 113.213 ± 15.650 | <0.001 | <0.001 |
| WEIGHT | 73.392 ± 16.968 | 81.179 ± 19.802 | 84.484 ± 21.349 | 88.380 ± 24.382 | <0.001 | <0.001 |
| RACE |  |  |  |  | <0.001 | - |
| 1 | 288 (9.979%) | 463 (16.043%) | 545 (18.884%) | 530 (18.358%) |  |  |
| 2 | 194 (6.722%) | 296 (10.256%) | 308 (10.672%) | 350 (12.123%) |  |  |
| 3 | 1070 (37.076%) | 1134 (39.293%) | 1112 (38.531%) | 1237 (42.847%) |  |  |
| 4 | 929 (32.190%) | 628 (21.760%) | 615 (21.310%) | 520 (18.012%) |  |  |
| 5 | 405 (14.033%) | 365 (12.647%) | 306 (10.603%) | 250 (8.660%) |  |  |
| SEX |  |  |  |  | <0.001 | - |
| 1 | 1708 (59.182%) | 1593 (55.198%) | 1374 (47.609%) | 982 (34.015%) |  |  |
| 2 | 1178 (40.818%) | 1293 (44.802%) | 1512 (52.391%) | 1905 (65.985%) |  |  |
| AGE |  |  |  |  | <0.001 | - |
| 1 | 2022 (70.062%) | 1268 (43.936%) | 870 (30.146%) | 577 (19.986%) |  |  |
| 2 | 584 (20.236%) | 903 (31.289%) | 823 (28.517%) | 639 (22.134%) |  |  |
| 3 | 280 (9.702%) | 715 (24.775%) | 1193 (41.337%) | 1671 (57.880%) |  |  |
| EDUCATIONAL\_LEVEL |  |  |  |  | <0.001 | - |
| 1 | 424 (14.692%) | 581 (20.132%) | 769 (26.646%) | 933 (32.317%) |  |  |
| 2 | 592 (20.513%) | 658 (22.800%) | 728 (25.225%) | 704 (24.385%) |  |  |
| 3 | 1662 (57.588%) | 1597 (55.336%) | 1359 (47.089%) | 1231 (42.639%) |  |  |
| 9 | 208 (7.207%) | 50 (1.733%) | 30 (1.040%) | 19 (0.658%) |  |  |
| MARITAL\_STATUS |  |  |  |  | <0.001 | - |
| 1 | 1496 (51.836%) | 1836 (63.617%) | 1856 (64.310%) | 1611 (55.802%) |  |  |
| 2 | 350 (12.128%) | 534 (18.503%) | 647 (22.419%) | 931 (32.248%) |  |  |
| 3 | 831 (28.794%) | 467 (16.182%) | 353 (12.231%) | 327 (11.327%) |  |  |
| 9 | 209 (7.242%) | 49 (1.698%) | 30 (1.040%) | 18 (0.623%) |  |  |
| PIR |  |  |  |  | <0.001 | - |
| 1 | 463 (16.043%) | 463 (16.043%) | 506 (17.533%) | 647 (22.411%) |  |  |
| 2 | 992 (34.373%) | 1075 (37.249%) | 1170 (40.541%) | 1226 (42.466%) |  |  |
| 3 | 1159 (40.159%) | 1084 (37.561%) | 921 (31.913%) | 689 (23.866%) |  |  |
| 9 | 272 (9.425%) | 264 (9.148%) | 289 (10.014%) | 325 (11.257%) |  |  |
| BMI |  |  |  |  | <0.001 | - |
| 1 | 1711 (59.286%) | 845 (29.330%) | 541 (18.765%) | 293 (10.167%) |  |  |
| 2 | 820 (28.413%) | 1121 (38.910%) | 1045 (36.247%) | 797 (27.654%) |  |  |
| 3 | 355 (12.301%) | 915 (31.760%) | 1297 (44.988%) | 1792 (62.179%) |  |  |
| ALCOHOL\_CONSUMPTION |  |  |  |  | <0.001 | - |
| 0 | 292 (10.118%) | 452 (15.662%) | 615 (21.310%) | 773 (26.775%) |  |  |
| 1 | 1820 (63.063%) | 1681 (58.247%) | 1462 (50.658%) | 1260 (43.644%) |  |  |
| 2 | 313 (10.845%) | 310 (10.742%) | 294 (10.187%) | 179 (6.200%) |  |  |
| 9 | 461 (15.974%) | 443 (15.350%) | 515 (17.845%) | 675 (23.381%) |  |  |
| SMOKING |  |  |  |  | <0.001 | - |
| 0 | 1733 (60.049%) | 1603 (55.544%) | 1500 (51.975%) | 1566 (54.243%) |  |  |
| 1 | 456 (15.800%) | 670 (23.216%) | 830 (28.760%) | 828 (28.680%) |  |  |
| 2 | 694 (24.047%) | 609 (21.102%) | 555 (19.231%) | 488 (16.903%) |  |  |
| 9 | 3 (0.104%) | 4 (0.139%) | 1 (0.035%) | 5 (0.173%) |  |  |
| HBP |  |  |  |  | <0.001 | - |
| 0 | 2325 (80.561%) | 1812 (62.786%) | 1521 (52.703%) | 1099 (38.067%) |  |  |
| 1 | 561 (19.439%) | 1074 (37.214%) | 1365 (47.297%) | 1788 (61.933%) |  |  |
| DIABETES |  |  |  |  | <0.001 | - |
| 0 | 2763 (95.738%) | 2528 (87.595%) | 2260 (78.309%) | 1865 (64.600%) |  |  |
| 1 | 123 (4.262%) | 358 (12.405%) | 626 (21.691%) | 1022 (35.400%) |  |  |
| CHD |  |  |  |  | <0.001 | - |
| 0 | 2654 (91.961%) | 2750 (95.288%) | 2710 (93.902%) | 2618 (90.682%) |  |  |
| 1 | 22 (0.762%) | 84 (2.911%) | 130 (4.505%) | 229 (7.932%) |  |  |
| 9 | 210 (7.277%) | 52 (1.802%) | 46 (1.594%) | 40 (1.386%) |  |  |
| SLEEP\_DURATION |  |  |  |  | <0.001 | - |
| 1 | 902 (31.254%) | 915 (31.705%) | 862 (29.868%) | 830 (28.750%) |  |  |
| 2 | 1535 (53.188%) | 1542 (53.430%) | 1522 (52.737%) | 1449 (50.191%) |  |  |
| 3 | 443 (15.350%) | 419 (14.518%) | 487 (16.875%) | 591 (20.471%) |  |  |
| 9 | 6 (0.208%) | 10 (0.347%) | 15 (0.520%) | 17 (0.589%) |  |  |
| OSA |  |  |  |  | <0.001 | - |
| 0 | 1795 (62.197%) | 1421 (49.238%) | 1332 (46.154%) | 1270 (43.990%) |  |  |
| 1 | 1091 (37.803%) | 1465 (50.762%) | 1554 (53.846%) | 1617 (56.010%) |  |  |

表中结果:
Mean+SD / N(%)
P值\*: 如是连续变量，用Kruskal Wallis秩和检验得出, 如计数变量有理论数<10，用Fisher精确概率检验得出.
此表用易侕统计软件 (www.empowerstats.com) 和R软件生成，生成日期： 2024-10-13
