# Supplementary material for: Non-linear association between weight-adjusted-waist index and obstructive sleep apnea: a cross-sectional study from the NHANES (2005–2008 to 2015–2020)
Source: Front Public Health. 2025 Mar 25;13:1546597. doi: 10.3389/fpubh.2025.1546597 (PMC11975944; doi:10.3389/fpubh.2025.1546597)
Supplement: Supplementary file 2 [file Data_Sheet_1.zip › Raw/table2/WWI quartile/20052020_10_tbl/20052020_10_tbl.htm]

## 单因素分析

Outcome: OSA
Exposure: WWI quartile
Adjust for: RACE SEX AGE
svy.DSN<-svydesign(id=~SDMVPS\_U, strata=~SDMVSTR\_A,weights=~WTSAF2Y\_R, data=WD,nest=TRUE)

|  |  |  |
| --- | --- | --- |
| Outcome: OSA | (N) % (95%CI) | OR (95%CI) P-value |
| WWI quartile |  |  |
| Q1 | (2508) 37.060 (34.320 ,39.800) | Ref. |
| Q2 | (2562) 50.473 (48.110 ,52.836) | 1.734 (1.493, 2.012) <0.0001 |
| Q3 | (2587) 55.425 (52.540 ,58.311) | 2.309 (1.956, 2.726) <0.0001 |
| Q4 | (2588) 57.105 (54.425 ,59.785) | 2.890 (2.400, 3.480) <0.0001 |

Data in table:
N: Number of observed
 % (95%CI): survey-weighted percentage (95% CI)
For
OSA
: survey-weighted OR (95%CI) p-value
Created by EmpowerStats (www.empowerstats.com) and R on 2024-10-13
