# Supplementary figures and images for: Non-linear association between weight-adjusted-waist index and obstructive sleep apnea: a cross-sectional study from the NHANES (2005–2008 to 2015–2020)
Source: Front Public Health. 2025 Mar 25;13:1546597. doi: 10.3389/fpubh.2025.1546597 (PMC11975944; doi:10.3389/fpubh.2025.1546597)

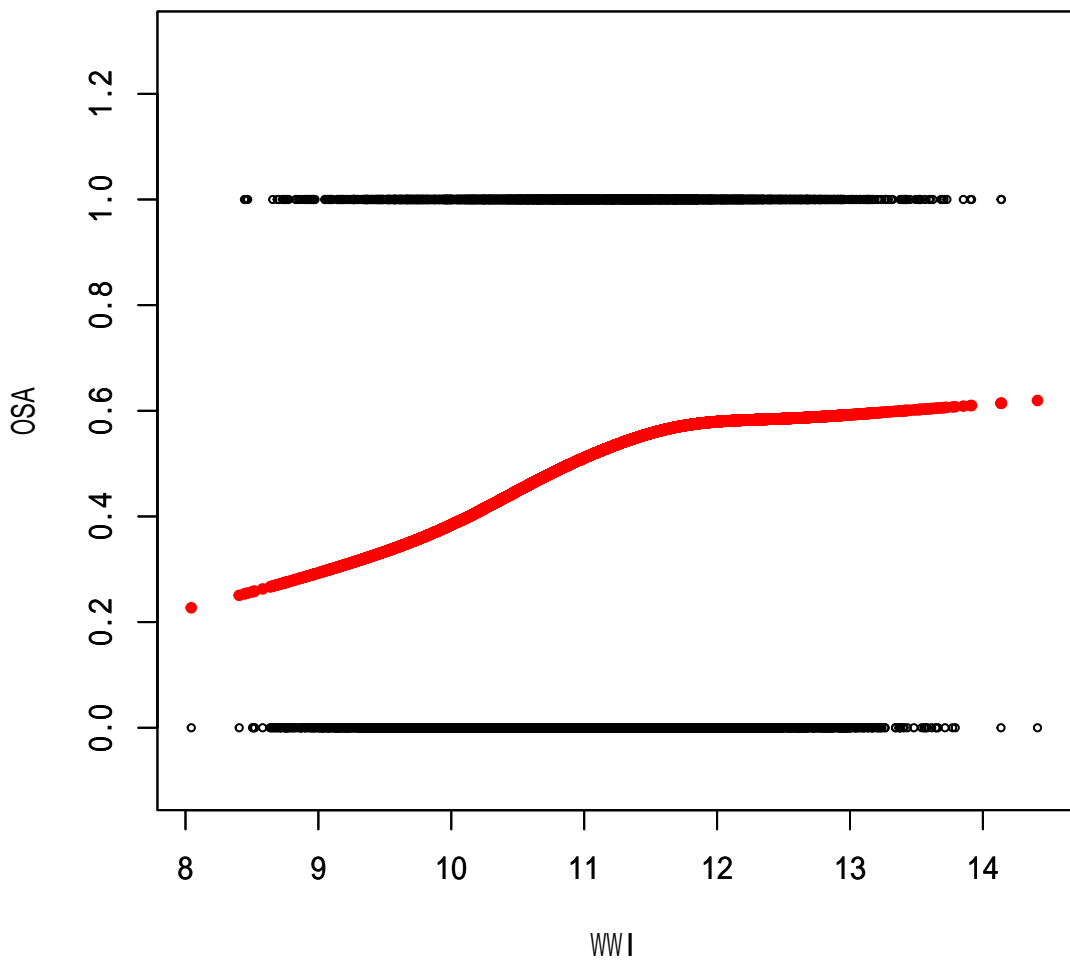

Supplement: Supplementary file 2 [file Data_Sheet_1.zip › Raw/Figure2/20052020_31_tbl/20052020_31_tbl_OSA_WWI_scatter.pdf]

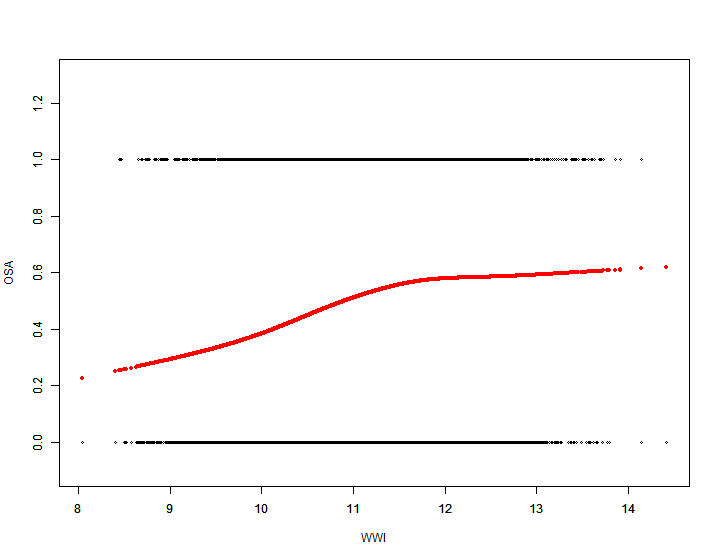

Supplement: Supplementary file 2 [file Data_Sheet_1.zip › Raw/Figure2/20052020_31_tbl/20052020_31_tbl_OSA_WWI_scatter.png]

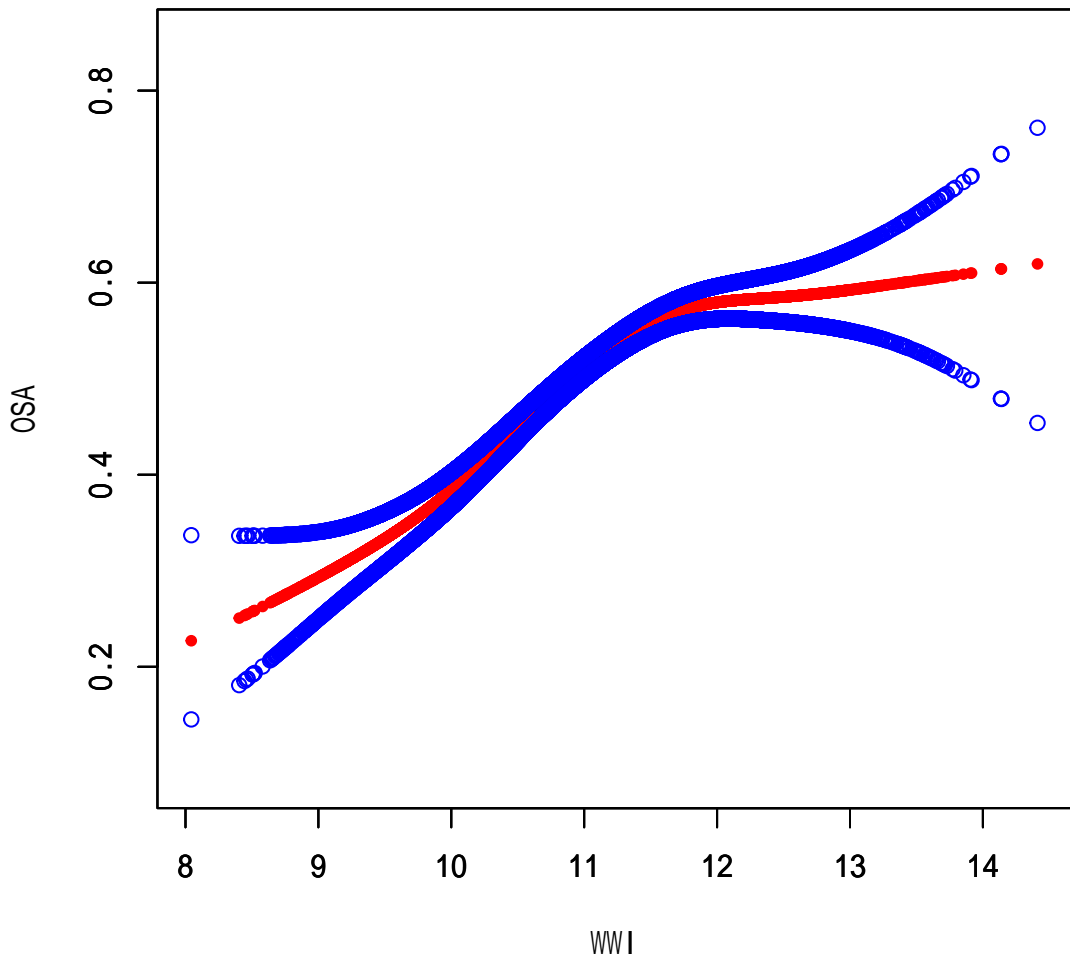

Supplement: Supplementary file 2 [file Data_Sheet_1.zip › Raw/Figure2/20052020_31_tbl/20052020_31_tbl_OSA_WWI_smooth.pdf]

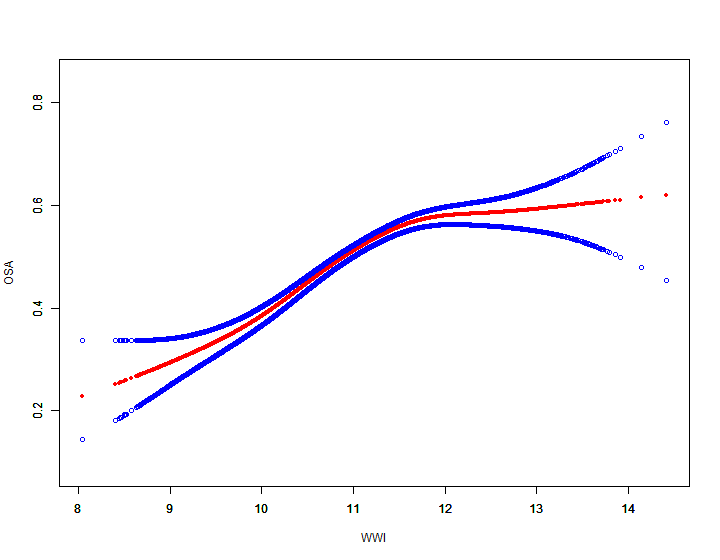

Supplement: Supplementary file 2 [file Data_Sheet_1.zip › Raw/Figure2/20052020_31_tbl/20052020_31_tbl_OSA_WWI_smooth.png]

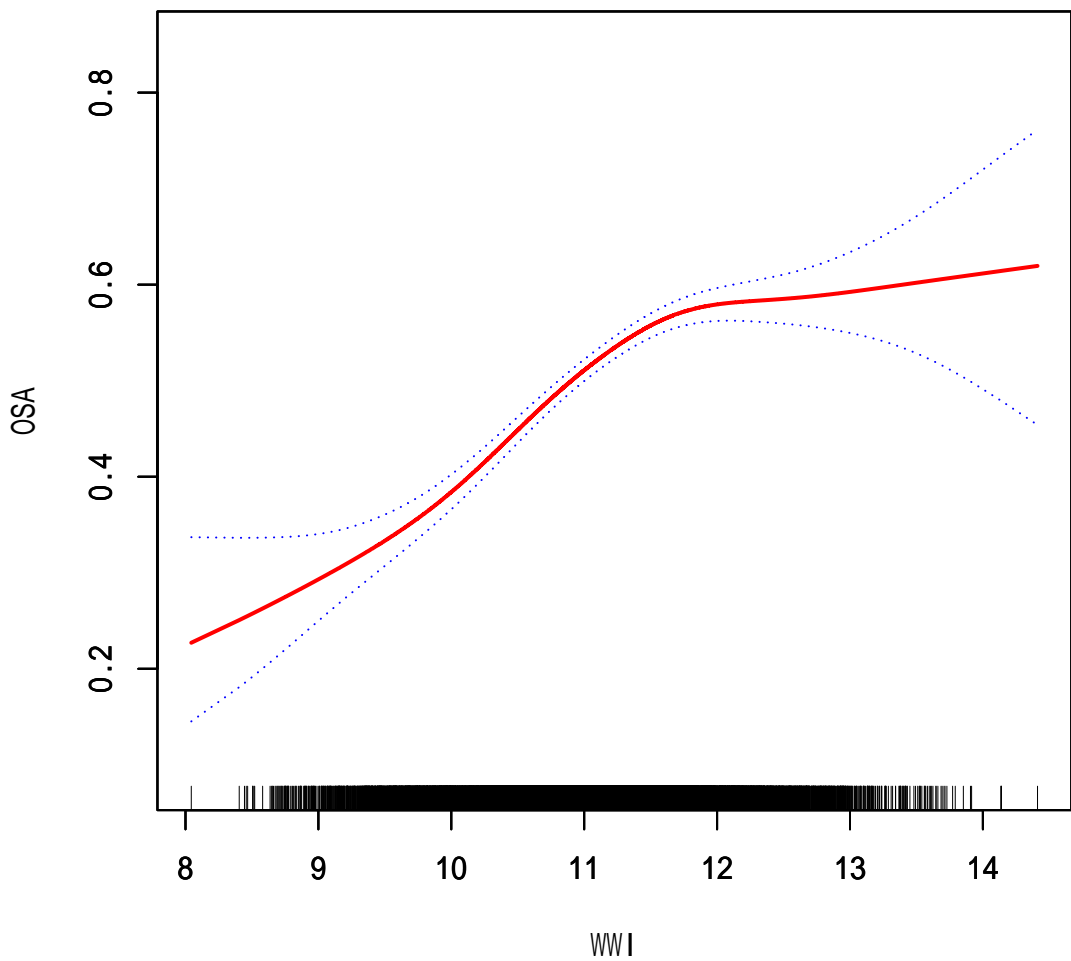

Supplement: Supplementary file 2 [file Data_Sheet_1.zip › Raw/Figure2/20052020_31_tbl/20052020_31_tbl_OSA_WWI_smooth1.pdf]

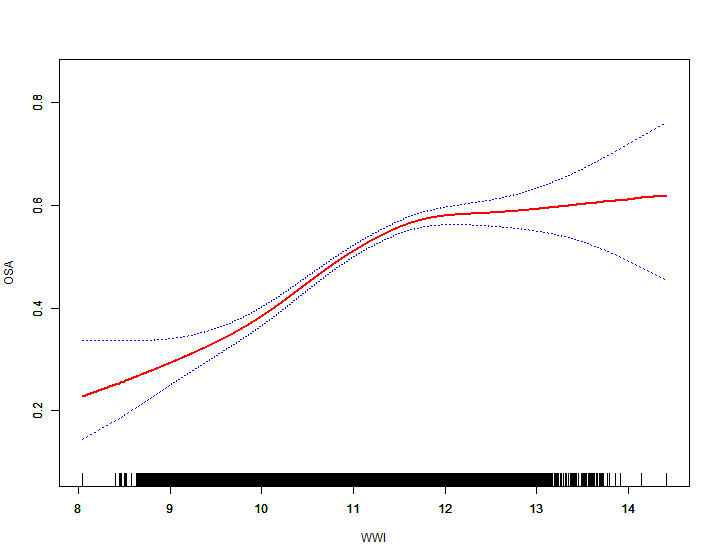

Supplement: Supplementary file 2 [file Data_Sheet_1.zip › Raw/Figure2/20052020_31_tbl/20052020_31_tbl_OSA_WWI_smooth1.png]

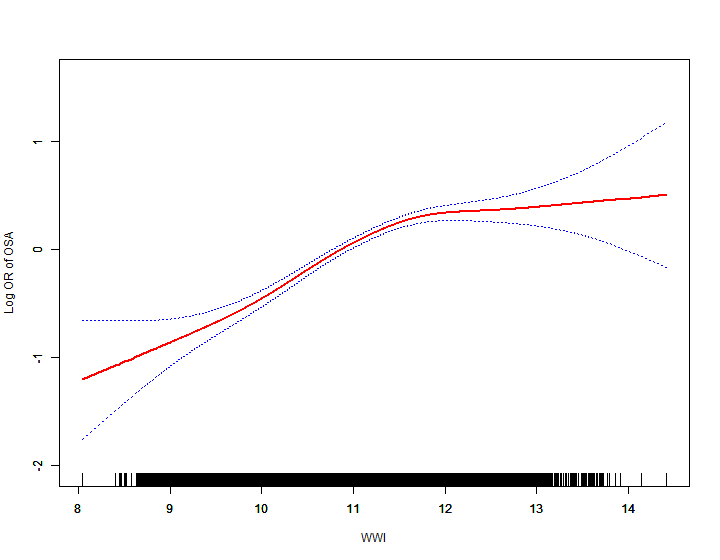

Supplement: Supplementary file 2 [file Data_Sheet_1.zip › Raw/Figure2/20052020_31_tbl/20052020_31_tbl_OSA_WWI_smooth2.png]

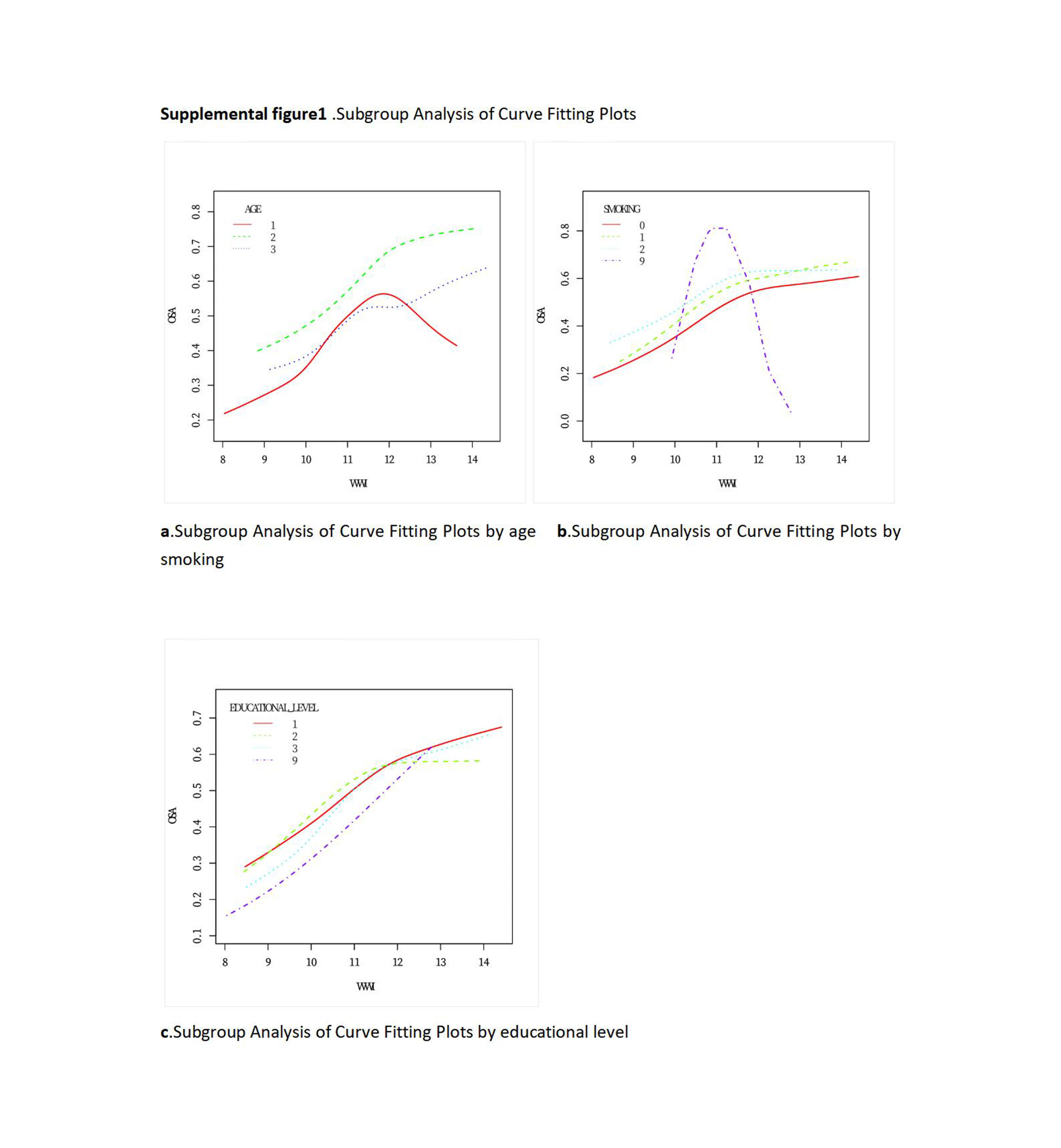

Supplement: Supplementary file 3 [file Image_1.jpeg]
